# Development of potent *in vivo* mutagenesis plasmids with broad mutational spectra

**DOI:** 10.1038/ncomms9425

**Published:** 2015-10-07

**Authors:** Ahmed H. Badran, David R. Liu

**Affiliations:** 1 Department of Chemistry and Chemical Biology, Harvard University, Cambridge, Massachusetts 02138, USA; 2 Howard Hughes Medical Institute, Harvard University, Cambridge, Massachusetts 02138, USA

## Abstract

Methods to enhance random mutagenesis in cells offer advantages over *in vitro* mutagenesis, but current *in vivo* methods suffer from a lack of control, genomic instability, low efficiency and narrow mutational spectra. Using a mechanism-driven approach, we created a potent, inducible, broad-spectrum and vector-based mutagenesis system in *E. coli* that enhances mutation 322,000-fold over basal levels, surpassing the mutational efficiency and spectra of widely used *in vivo* and *in vitro* methods. We demonstrate that this system can be used to evolve antibiotic resistance in wild-type *E. coli* in <24 h, outperforming chemical mutagens, ultraviolet light and the mutator strain XL1-Red under similar conditions. This system also enables the continuous evolution of T7 RNA polymerase variants capable of initiating transcription using the T3 promoter in <10 h. Our findings enable broad-spectrum mutagenesis of chromosomes, episomes and viruses *in vivo*, and are applicable to both bacterial and bacteriophage-mediated laboratory evolution platforms.

## Abstract (web summary)

Random DNA mutagenesis provides genetic diversity both in nature and the laboratory. Here, Badran and Liu present a potent, inducible, broad-spectrum and vector-based mutagenesis system in *E. coli* that surpasses the mutational efficiency and spectra of the most widely used *in vivo* and *in vitro* mutagenesis methods.

## Graphical abstract

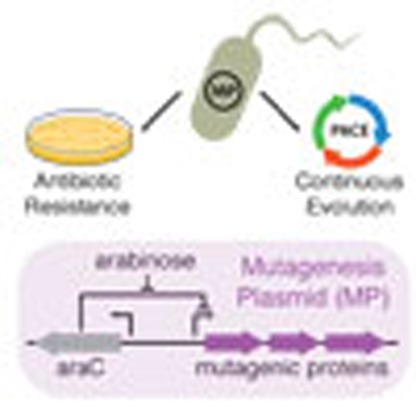

Access to new mutations drives both natural and laboratory evolution. Native biological mutation rates are modest, occurring at frequencies of approximately one mutation per billion replicated DNA bases in most eukaryotes and prokaryotes^1^. Laboratory evolution methods must increase this mutation rate to accelerate the discovery of biomolecules with desired properties on a practical timescale. In addition to mutation rate, mutational spectrum is also a crucial component of the genetic diversity that fuels evolution. Access to diverse amino-acid substitutions during evolution is enhanced by more complete coverage of the 12 possible mutation types.

Several systems to enhance mutational efficiency and broaden mutational spectra have been developed for use in laboratory evolution efforts^2^. *In vitro* mutagenesis through error-prone PCR, site-saturation mutagenesis or DNA shuffling has become the standard approach to introduce diversity into genes of interest. Whereas *in vitro* mutagenesis methods enable control of mutation rate and mutational spectrum, *in vivo* mutagenesis methods enable mutation and selection cycles to be coupled, bypass transformation efficiency bottlenecks that frequently limit the size of gene populations that can be accessed following *in vitro* mutagenesis, and avoid labour-intensive library creation, cloning and manipulation steps^3^. The difficulty of tuning mutagenic load and spectrum in live cells, however, has prevented the development of safe, effective *in vivo* mutagenesis methods that can rival or exceed the efficiency and mutational spectra of state-of-the-art *in vitro* methods.

The most commonly used *in vivo* mutagenesis methods include chemical mutagens, nucleobase analogues, ultraviolet light and hypermutator strains^4^. Chemical mutagens yield narrow mutagenic spectra and are potent human carcinogens. Ultraviolet radiation is known to generate a wide mutation spectrum with little sequence preference, but with low potency that is limited by cellular toxicity. Perhaps the most widespread method for *in vivo* mutagenesis has been the use of hypermutator strains such as XL1-Red^5^ that have been engineered to have higher mutation rates through the deletion or modification of genes involved in DNA replication and repair. These strains suffer from numerous drawbacks, however, including modest mutational potency (*vide infra*), moderately biased mutational preference^6^, poor transformation efficiency, slow growth rate and difficulty of modification when additional genetic manipulations are required. In addition, the rate of mutagenesis is not controllable, and mutagenesis generally must be separated from other steps such as selection or screening due to the instability or poor growth of the strain. Loeb and co-workers previously described an elegant system that uses a low-fidelity *Escherichia coli* DNA Pol I (LF-Pol I) to increase *in vivo* mutagenesis efficiency with a wide scope of mutational types^7^. Unfortunately, this method is restricted to Pol I temperature-sensitive strains and mutates only vectors carrying ColE1-related origins of replication, with mutation rate being highly dependent on distance from the ColE1 origin^7^,^8^.

An ideal *in vivo* mutagenesis method should offer (i) a broad mutagenesis spectrum, (ii) a mutation rate that can be very high but is easily modulated by the researcher and (iii) entirely episomal encoding so that it can be applied to virtually any *E. coli* strain. Here we describe the development of a general *in vivo* mutagenesis method that meets these criteria. We generate a series of vectors that enable robust chromosomal, episomal and viral DNA mutagenesis in a strain-independent manner. Importantly, this system outperforms commonly used *in vivo* mutator and chemical mutagenesis methods in mutational efficiency and spectrum, and can even achieve mutational potency and spectrum comparable to that of state-of-the-art *in vitro* mutagenesis methods. We apply this system to two directed evolution case studies in bacteria and bacteriophage, validating their ability to access biomolecule variants with novel properties with greater effectiveness than previously described methods. Collectively, this system substantially advances *in vivo* mutagenesis capabilities and increases the effectiveness of laboratory evolution efforts.

## Results

### Mutagenesis plasmid minimization

Bacteria control the rate of chromosomal substitutions through a series of overlapping mechanisms that can be subdivided into three main pathways: proofreading (reduces mutation rate by a factor of ∼10^2^ substitutions per base pair (bp) per generation), mismatch repair (reduces mutation rate by ∼10^3^ substitutions per bp per generation) and base selection (reduces mutation rate by ∼10^5^ substitutions per bp per generation)^9^ (Supplementary Fig. 1). These redundant replication maintenance mechanisms collectively account for the basal substitution rate of bacterial chromosomal DNA of ∼10^−9^ to 10^−10^ substitutions per bp per generation^9^. On the basis of prior knowledge of dominant mutators alleles that interfere with DNA replication fidelity, we sought to design a series of small-molecule inducible mutagenesis plasmids (MPs) that offer broad mutational spectra and high levels of mutagenesis in bacterial cells.

We recently reported the development and application of an MP for *in vivo* mutagenesis during phage-assisted continuous evolution (PACE)^10^. This MP increases the mutation rate ∼100-fold above the basal *E. coli* mutation rate through the arabinose-induced expression of *dnaQ926*, a dominant-negative variant of the *E. coli* DNA Pol III proofreading domain. This plasmid additionally provides *umuD′*, *umuC* and *recA730*, which together enable *in vivo* translesion mutagenesis employing ultraviolet light or chemical mutagens. This MP (designated MP1) results in a substitution rate of 7.2 × 10^−5^ and 5.4 × 10^−8^ substitutions per bp per generation for M13 phage and *E. coli*, respectively^10^,^11^. This mutation rate, however, is still several orders of magnitude below the mutation rates provided by conventional *in vitro* mutagenesis techniques^12^.

**Figure 1:**
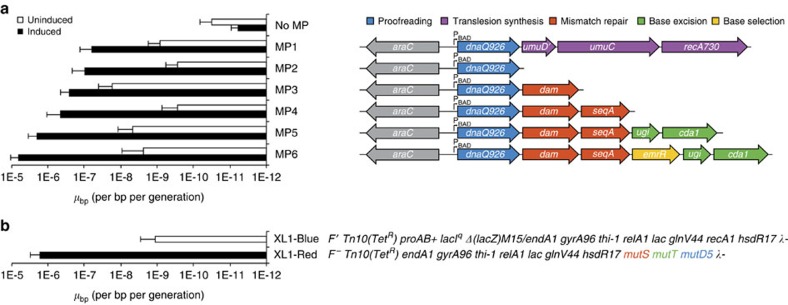
MP design and effect on mutation rate in bacteria. The mutator genes tested are colour-coded to indicate the canonical pathway being disrupted through the overexpression (**a**) or deletion (**b**) of that gene. All MPs express mutator genes from the *E. coli* arabinose-inducible P_BAD_ promoter, with each gene preceded by a ribosome-binding site to enable translation from a single transcript. For **a**, the mutagenesis rate *μ*_bp_ (substitutions per base pair of the *E. coli* genome per generation) was calculated using rifampin resistance under uninduced (25 mM glucose, white bars) and induced (25 mM glucose+25 mM arabinose, black bars). For **b**, the rifampin resistance of XL1-Blue (white bar) and XL1-Red (black bar) was used to calculate *μ*_bp_. In all cases, all known 77 rifampin-resistant *rpoB* alleles were used to calculate the mutation rate. Error bars denote the standard deviation of at least three biological replicates.

Since we sought to avoid the use of exogenous mutagens, we first minimized MP1 by removing *umuD′*, *umuC* and *recA730* from MP1 to yield MP2 carrying only *dnaQ926*, and observed mutation rates in the absence of mutagens to be modestly improved compared with MP1 through a rifampin-resistance assay using the nearly wild-type *E. coli* MG1655 Δ*recA::apra* (Supplementary Table 1), enabling an average of 9.9 × 10^−8^ substitutions per bp per generation (Fig. 1a). Since *dnaQ926* abrogates the proofreading component of DNA replication, we began assessing additional genes that when expressed from the MP can further enhance mutation rate.

### DNA methylation state manipulation enhances mutagenesis

Mismatch repair reduces the error rate of bacterial DNA replication by a factor of up to ∼10^3^ (ref. 9). Specialized proteins monitor (MutSL) and excise (MutH) mismatched nucleotides following DNA replication, using DNA methylation (Dam) to indicate the parental strand and thus the basis for correction. Not surprisingly, the deletion of *mutS*, *mutL* or *mutH*, and the overexpression of *dam* are known to have a strong mutator effect due to impaired mismatch repair^13^.

We added wild-type *dam* downstream of *dnaQ926* on the same arabinose-inducible expression cassette to yield MP3, and observed increased mutagenesis potency but also increased uninduced background mutagenesis, likely due to a strong cryptic σ^70^ promoter at the 3′ end of *dnaQ926* (Supplementary Fig. 2). Overall, MP3 resulted in a 3-fold mutagenesis potency increase in the presence of arabinose relative to MP2 but >60-fold increase in background mutagenesis, greatly reducing the dynamic range of induced mutagenesis to only 11-fold (Fig. 1a).

To restore dynamic range, we installed the gene encoding the hemimethylated GATC-binding domain SeqA downstream of *dam*. Low-level expression of *seqA* is known to delay Dam methylation of hemimethylated GATC sequences^14^ and induce positive supercoils in chromosomal and episomal DNA^15^, which may reduce MP gene transcription in the absence of arabinose. Conversely, high-level *seqA* expression results in a potent mutator response^16^ and induction of negative supercoils in chromosomal and episomal DNA^15^, potentially increasing global gene transcription, including that of the MP, on induction with arabinose. Indeed, the MP carrying *dnaQ926*, *dam* and *seqA* (MP4) resulted in >60-fold reduced background and 2-fold improved mutagenesis potency in the presence of arabinose, representing a cumulative >100-fold improvement in dynamic range, relative to MP3 (Fig. 1a). In agreement with previous reports showing that dominant-negative *dnaQ* mutants partially saturate the mismatch-repair response^17^, dominant-negative variants of *mutS*^16^,^18^, *mutL*^19^,^20^ or *mutH*^20^ with *dnaQ926* had no additional effect on mutagenesis (Supplementary Tables 2 and 3). Cumulatively, expression of *dam* and *seqA* from MP4 is sufficient to disrupt the mismatch-repair response, enabling an average of 4.4 × 10^−7^ substitutions per bp per generation.

### Cytosine deamination and reduced base-excision repair

Overexpression of the catalytic domains of several cytidine deaminases in *E. coli* has been shown to have a mutagenic effect, resulting in primarily C→T transitions through a deoxyuracil intermediate^21^. The cytidine deaminase CDA1 from *Petromyzon marinus* is reported to promote the mutation of prokaryotic and eukaryotic genomic DNA^21^. Impairing uracil–DNA glycosylase (Ung) synergizes with the effect of deaminases and can enhance mutagenesis through disruption of the native uracil-excision repair pathway^21^ (Supplementary Fig. 1). Two natural protein inhibitors of Ung, Ugi and p56 (ref. 22), inhibit Ung through mimicry of structural and electronic features of uracil-containing DNA^22^. As the p56:Ung interaction has been shown to be less stable than the Ugi:Ung interaction^22^, we proceeded with Ugi.

We placed *ugi* and *cda1* downstream of *dnaQ926*, *dam* and *seqA* to yield MP5, and observed a 5-fold mutagenesis potency increase under induced conditions, and an 18-fold increase in background mutagenesis, compared with MP4 (Fig. 1a). This background increase was caused by two predicted σ^70^ promoters at the 3′ end of the *seqA* open reading frame, resulting in constitutive *ugi* and *cda1* transcription (Supplementary Fig. 3). We considered this background mutation rate for MP5 to be acceptable, as it was only ∼5-fold higher than the starting MP1. Alternative cytosine deaminases, including rat APOBEC1 and human AID, generally resulted in weaker effects on mutation rate than CDA1, in agreement with previous reports (CDA1>>AID≈APOBEC1) (ref. 21) (Supplementary Tables 2 and 3). Overall, MP5 yielded 2.0 × 10^−6^ substitutions per bp per generation, a 31-fold increase in mutation rate relative to MP1 (Supplementary Tables 2 and 3).

### Impairing mutagenic nucleobase export

Two major determinants of base selection during DNA replication are the catalytic alpha subunit of *E. coli* DNA Pol III and the intracellular concentration of dNTPs (Supplementary Fig. 1). Mutations affecting the former are generally not viable or exert a mutator effect through reduced affinity to the proofreading domain DnaQ^23^, whereas perturbations affecting the latter are generally more tolerated and can be modified to affect the mutational spectrum^24^. We screened a number of proteins that are known to compromise intracellular dNTP pools, and found expression of the *emrR* transcriptional repressor to be the most promising (Fig. 1a; Supplementary Tables 2 and 3).

Derepression by EmrR results in upregulation of *emrAB*, which produces a multidrug efflux pump responsible for antibiotic resistance and the putative export of mutagenic nucleobase intermediates^25^. In an unbiased screen, *emrR* overexpression was found to have a potent mutator effect^16^, presumably as a consequence of retaining these mutagenic nucleobases within the cell. To decrease background mutagenesis compared with MP5, we placed the *emrR* cassette between *seqA* and *ugi*, thereby disrupting the strong predicted σ^70^ promoter, yielding MP6. MP6 exhibited twofold lower background mutagenesis, while improving the overall mutator effect under induced conditions by threefold (Fig. 1a).

### Features of the MP6 mutagenesis system

We chose MP6 for in-depth characterization because it offered the highest mutagenic potency with acceptable levels of toxicity (<100-fold reduction in cell viability under maximal MP induction compared with the uninduced control). Efforts to increase the expression level of the six key genes in MP6 resulted in substantially higher toxicity or reduced potency (Supplementary Tables 2 and 3 and Supplementary Note 1). When induced, MP6 results in a 322,000-fold increase in mutation rate of chromosomal DNA over that of wild-type *E. coli*, and a 100-fold increase in mutation rate relative to that of MP1. Induced MP6 results in an average of 6.2 × 10^−6^ substitutions per bp per generation, representing to our knowledge the most potent inducible and genetically encodable mutagenesis method in bacteria reported to date (Supplementary Table 4). This potency compares favourably with overexpression of *PolB (D156A)* (*μ*_bp_=3.2 × 10^−6^), *dnaQ926* (*μ*_bp_=2.4 × 10^−6^) or *mutD5* (*μ*_bp_=3.9 × 10^−7^) (Supplementary Table 4). We note that most previously published mutagenic constructs are not inducible, negatively affect host viability and require overexpression of the mutagenic protein. In contrast, the MPs described here rely on low-level expression of multiple genes, thereby affecting multiple pathways and enabling broad mutagenic spectra.

Additional permutations of this design or the inclusion of alternative mutators impaired overall mutation rate, or strongly decreased host viability, as evidenced by the characteristics of 80 candidate MPs with mutation rates spanning five orders of magnitude (Supplementary Figs 4–6 and Supplementary Tables 2 and 3). We observed that a loss of bacterial viability proportional to mutagenic potency beyond a mutation rate of ∼4 × 10^−7^ substitutions per bp per generation, corresponding to an average of ∼1.9 substitutions per genome per generation. Given that ∼10–30% of the *E. coli* genome has been estimated to be essential^26^, this mutation rate threshold corresponds to ∼0.2–0.6 mutations in an essential gene/generation.

**Figure 2:**
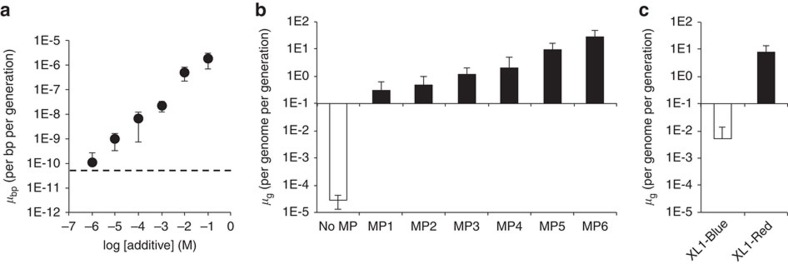
Features of the MP system. (**a**) The dynamic range of MP6 was evaluated using increasing concentrations of arabinose (black circles) in the presence of 25 mM glucose in all cases. Treatment with 200 mM glucose only (dotted line) showed low mutagenesis under identical conditions. Comparison of the mutation rate under induced and uninduced conditions reveals a 35,000-fold dynamic range. Using the number of known unique mutations found in rifampin-resistant *rpoB* alleles (77 sites), the average number of substitutions per *E. coli* genome per generation (*μ*_g_) was calculated for (**b**) MG1655 Δ*recA::apra* without an MP (white bar) versus carrying MP1-6 under induced conditions (black bars), and (**c**) XL1-Blue (white bar) versus XL1-Red (black bar). Error bars denote the standard deviation of at least three biological replicates.

To test the inducibility of MP6, we increased the concentration of glucose from 25 to 200 mM during the transformation and growth stages before induction to maximize catabolite repression of the arabinose-inducible promoter. Under these modified conditions, induction of log-phase cultures of MG1655 Δ*recA::apra* carrying MP6 with increasing concentrations of arabinose resulted in a 35,000-fold range of mutational potency between 10 μM and 100 mM arabinose (Fig. 2a). Despite this strong induction effect, MP6-carrying cultures maintained low levels of mutagenesis when suppressed with 200 mM glucose (Fig. 2a).

To further evaluate MP6, we compared its performance with that of the most commonly used *in vivo* mutagenesis strain, XL1-Red (Agilent Technologies). XL1-Red is deficient in proofreading (*mutD5*), mismatch-repair (*mutS*) and base-excision (*mutT*) activities, resulting in high levels of substitutions in chromosomal and episomal DNA^5^. However, the strain grows very slowly, is difficult to manipulate genetically, has poor transformation efficiency and produces a fairly narrow mutagenic spectrum^6^. Using the rifampin-resistance assay, we found that XL1-Red results in 8 substitutions per genome per generation, while MP6 in the induced state produces an average of 29 substitutions per genome per generation, a ∼4-fold higher mutation rate (Fig. 2b,c). The uninduced state of MP6 yields similar background mutagenesis levels (0.01 substitutions per genome per generation) as the non-mutagenic related strain XL1-Blue (0.005 substitutions per genome per generation) (Fig. 1a, b). Together, these results establish that MP6 enables mutagenesis levels exceeding that of the most commonly used mutator *E. coli* strain, and offers the ability to control mutation rate with an exogenous small molecule.

### MP6 augments M13 bacteriophage mutagenesis

To further characterize MP1, MP4 and MP6, we assessed their impact on the mutagenesis of bacteriophage DNA, a common component of laboratory screening and evolution efforts. We measured the mutagenesis rate of M13 phage in host cells harbouring a variety of MP variants or in XL1-Red using a *lacZ* inactivation assay. Briefly, a *lacZ* cassette was integrated downstream of *geneIII* in the wild-type M13 genome to yield SP063, resulting in the production of blue plaques in the presence of an X-Gal analogue (Supplementary Fig. 7). Disruption of the *lacZ* cassette due to high mutagenesis reduces or ablates this blue-plaque phenotype, enabling the estimation of phage mutagenesis rates. We compared the ratio of white:blue plaques (*lacZ*^**-^ phenotype) using MP1, MP4, MP6 and XL1-Red.

**Figure 3:**
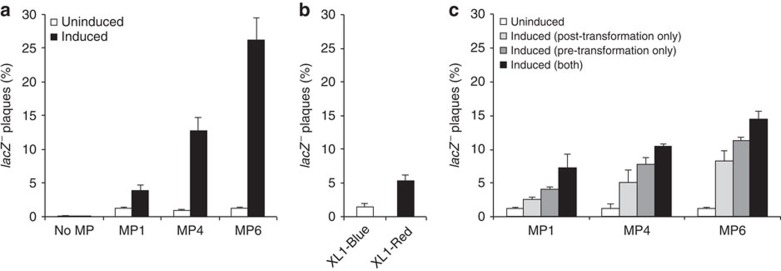
*In vivo* mutagenesis of M13 bacteriophage DNA. (**a**) S1030 cells (*F*′) carrying the indicated MPs were infected with M13 bacteriophage carrying a constitutive *lacZ* expression cassette (SP063), accompanied by the induction of the MP using arabinose, or suppression of the MP using glucose. (**b**) XL1-Blue or XL1-Red cells were transformed with purified SP063 DNA and recovered after overnight growth. (**c**) S1021 cells (identical to S1030, but lacking *F′*) carrying the indicated MPs were induced with arabinose or repressed with glucose for 2 h, transformed with purified SP063 DNA, and again induced with arabinose or repressed with glucose during recovery. For **a**–**c**, progeny phage from the overnight propagation were plaqued on S1030 cells and stained using the X-Gal analogue Bluo-Gal. The fraction of plaques that showed a white or light blue *lacZ*^*−*^ phenotype reflecting loss-of-function mutation(s) is shown. Error bars denote the standard deviation of at least three biological replicates.

Under conditions supporting phage inoculum expansion in overnight culture using S1030 cells (Supplementary Fig. 8 and Supplementary Table 1), we observed that the phage-borne *lacZ* inactivation rates scaled with MP potency, reaching up to 27% white plaques (representing mutant, inactive *lacZ* genes) after 18–24 h of culture with MP6 (Fig. 3a). As MP6 increases the rate of mutagenesis by ∼100-fold as compared with MP1, the expected M13 bacteriophage mutation rate is elevated to 7.2 × 10^−3^ substitutions per bp per generation, resulting in an average of 22 substitutions per genome per generation in the SP063 phage. This mutagenesis potency of 2.3 substitutions per kbp (0.23%) approaches the potency of the most commonly used *in vitro* mutagenesis methods, Mutazyme II (0.3–1.6%, Agilent Technologies).

To enable comparison to XL1-Red, which lacks an *F′* episome and thus cannot be infected by M13 phage particles but allows for the production of fully functional phage, purified SP063 dsDNA was transformed into S1021 (the *F*^*−*^ variant of S1030, Supplementary Table 1) cells carrying the aforementioned MPs (induced before or after SP063 transformation, or both; Supplementary Fig. 9) or XL1-Red. Whereas phage produced from transformed XL1-Red cells only yielded an average of 5% white plaques on S1030 cells (Fig. 3b), phage produced from the S1021 strain carrying MP6 yielded 15% white plaques and MP1 and MP4 yielded 7% and 10% white plaques, respectively, on S1030 cells (Fig. 3c). These results further demonstrate the greater mutational potency of MP6 compared with that of XL1-Red, and also highlight the strain flexibility enabled by using inducible, genetically encodable mutagenesis systems.

### Mutational spectra of designed MPs

**Figure 4:**
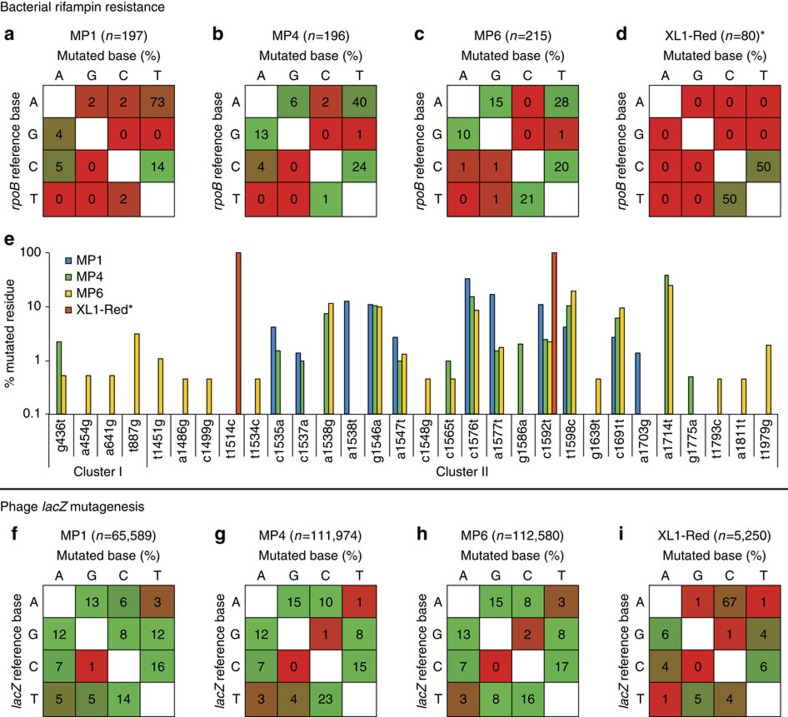
Mutagenic spectra of the MPs. (**a**–**d**) The *rpoB* locus of single rifampin-resistant colonies was amplified by PCR and sequenced in both clusters I (aa 451–754) and II (aa 84–401). (**e**) Identities of the rifampin-resistant *rpoB* alleles from MP1, MP4 or MP6 mutagenesis, or using XL1-Red. The MPs yielded a wide distribution of mutation types, with the diversity of alleles strongly correlating with MP potency. (**f**–**i**) SP063 phage containing a constitutive *lacZ* expression cassette was propagated on the indicated mutator strain under induced conditions, and mutations were analysed by high-throughput sequencing. In all cases, the number of sequenced mutations (*n*) is indicated for the MP and XL1-Red assays. *For **d** and **e**, all sequenced XL1-Red rifampin-resistant colonies carried the identical F505S/S531F *rpoB* genotype. aa, amino acid.

In addition to mutagenic potency, the distribution of mutation types is also important, as a narrow mutational spectrum limits the diversity of changes that can be accessed. To analyse the spectrum of produced mutations, we took advantage of previously reported distributions of rifampin-resistant *rpoB* mutants. Importantly, mutations covering each of the 12 possible transitions and transversions in the *rpoB* gene are known to endow *E. coli* with resistance to high levels of rifampin^27^. We assessed the spectra of MP1, MP4 and MP6 by sequencing rifampin-resistant *rpoB* alleles in mutated MG1655 Δ*recA::apra*, and compared the spectra of each with the *rpoB* mutation spectrum afforded by XL1-Red (Fig. 4a–d). MP1 yielded a narrow mutagenic spectrum strongly biased towards A:T→T:A transversions, a known side effect of using *recA730*-based mutators on genomic DNA^28^. Comparatively, the intermediate MP4 had a more uniform distribution, covering more transitions and a marked increase in G:C→A:T and A:T→G:C, a hallmark of mutagenesis methods that perturb the mismatch-repair response^29^. MP6 exhibited a wider spectrum still, with a more uniform distribution of transitions and transversions. The identities of the observed rifampin-resistant mutations are in agreement with previous studies (Fig. 4e)^27^. Notably, XL1-Red exclusively displayed two types of mutations, C:G→T:A and T:A→C:G transitions (Fig. 4d,e). This observation is consistent with previous reports describing the narrow mutational spectrum of XL1-Red^6^. Additional MP characterization the β-galactosidase (*lacZ)* reversion strains developed by Miller and co-workers^30^ revealed similar mutational spectra (Supplementary Fig. 10 and Supplementary Note 2).

To further characterize the mutagenic spectra, we propagated the *lacZ*-encoding phage SP063 using S1030 strains carrying MP1, MP4 and MP6, or transformed SP063 DNA directly into XL1-Red to produce progeny phage, and subjected the *lacZ* open reading frame from all the resultant phage to high-throughput DNA sequencing (Fig. 4f–i). The mutagenesis efficiency for all conditions was in agreement with that revealed by the other assays (Fig. 4a–i and Supplementary Fig. 10). The MPs generated broad mutagenic spectra in progeny phage, consistent with both *rpoB* single-clone sequencing and *lacZ* reversion assays, with the exception of MP1, which showed a more uniform distribution of mutation types using the phage assay and *lacZ* reversion assays than using *rpoB* sequencing. This discrepancy is likely a result of the MP1-encoded *recA730* allele, which selectively enhances the rate of A:T→T:A transversions in DNA of strains lacking *recA* (MG1655 Δ*recA::apra*), as it no longer competes for substrates with wild-type (CSH strains^30^, Supplementary Table 1) or reduced-activity *recA* mutants (S1021 and S1030, Supplementary Table 1) for function. Interestingly, the MP6 mutagenic spectrum shows an enhancement in C→T transitions with a concomitant decrease in T→C transitions, a hallmark of cytidine deaminase-mediated mutagenesis. Phage sequences produced from XL1-Red revealed a much narrower mutational spectrum, with a bias for A:T→C:G mutations. We further note that the number of mutations among the tested MPs does not agree with differences in mutagenesis potency, likely due to mutation accumulation in the bacteriophage genome, which would negatively affect propagation and thus mutation accumulation. Taken together, these results reveal that the MPs developed in this study can outperform the most widely used *in vivo* and *in vitro* mutagenesis techniques (Supplementary Fig. 11) both in mutagenic potency and mutational breadth.

### Evolution of antibiotic resistance using the designed MPs

To evaluate the impact of these MPs on laboratory evolutionary outcomes, we evolved antibiotic resistance in *E. coli*, as well as RNA polymerase substrate specificity changes in bacteriophage, using cells harbouring different MPs. First we tested the ability of MP1, MP4 or MP6 to evolve resistance of *E. coli* strain MG1655 Δ*recA::apra* to a number of commonly used antibiotics. Mid-log-phase cultures of MG1655 Δ*recA::apra* carrying MP1, MP4 or MP6 were induced for 18–21 h, then serially diluted and plated on agar plates without antibiotics, or with 5–100 μg ml^−1^ of carbenicillin, cephotaxime, fosfomycin, kanamycin, metronidazole, norfloxacin, rifampin, spectinomycin, streptomycin or tetracycline. The antibiotic concentrations used in all 10 cases were well above known minimum inhibitory concentration values (Supplementary Table 5).

**Figure 5:**
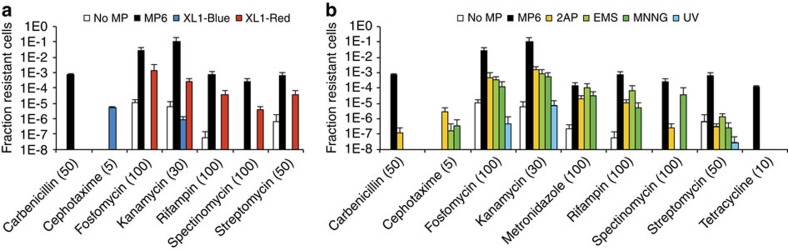
Comparison of MP6 and other mutagenesis approaches for the evolution of antibiotic resistance. (**a**) MG1655 Δ*recA::apra* cells with or without MP6 were grown for 18–21 h under induced conditions. XL1-Blue and XL1-Red were grown for 18–21 h. (**b**) MG1655 Δ*recA::apra* cells were treated using chemical mutagens as previously described^30^. In all cases, cultures were plated on the indicated antibiotics following overnight culture in the absence of any selection. The numbers in parenthesis indicate the antibiotic concentrations used in μg ml^−1^. The fraction of cells resistant to each antibiotic was calculated relative to the total number of cells on plates without antibiotics. Resistance to norfloxacin was not detected for any of the tested strains or treatments. XL1-Blue and XL1-Red are both inherently resistant to tetracycline and metronidazole, so no comparison is shown for either antibiotic. See Supplementary Table 6 for full antibiotic resistance data. Error bars denote the standard deviation of at least three biological replicates. UV, ultraviolet irradiation.

After only 18 h of growth on antibiotic-containing solid medium, large fractions of the bacterial population showed resistance to high concentrations of carbenicillin, fosfomycin, kanamycin, metronidazole, rifampin, spectinomycin, streptomycin and tetracycline (Fig. 5a and Supplementary Table 6). No resistant colonies were detected for cefotaxime or norfloxacin. The frequency of antibiotic resistance strongly correlated with MP potency (Fig. 5a). For example, we observed the evolution of resistance to high levels of kanamycin with no intermediate selection step using MP6, which enabled up to 11% of the total population to grow in the presence of 30 μg ml^−1^ kanamycin. In contrast, only 0.02 or 0.66% of the population survived this level of kanamycin when using MP1 or MP4, respectively (Fig. 5a).

We repeated these antibiotic-resistance evolution experiments to compare the performance of these MPs with those of the commonly used chemical mutagens^30^ ethyl methanesulfonate (EMS), methylnitronitrosoguanidine (MNNG) and 2-aminopurine (2AP), as well as ultraviolet irradiation, XL1-Red and XL1-Blue (Fig. 5a,b and Supplementary Table 6). Importantly, ultraviolet irradiation can only be used for short durations due to toxicity from random crosslinking of cellular components. As such, mutagenesis with ultraviolet irradiation typically constitutes a single round of mutagenesis, while chemical mutagens, hypermutator strains and mutagenesis plasmids enable continuous mutagenesis. MP6 outperformed all six of the other mutagenesis treatments or strains for all but one of the antibiotics. Cephotaxime resistance was not observed from use of any of the MPs, but was weakly detected from the chemical mutagens (Fig. 5b). We speculate that the known ability of all three of these chemical mutagens to greatly enhance G:C→A:T transitions may contribute to the evolution of cephotaxime resistance. This finding further suggests that a period of neutral drift or intermediate selection pressure may be needed to evolve complex phenotypes requiring mutations to multiple cellular components. Cumulatively, these results suggest that MP6 can rapidly generate strains with novel properties, outperforming several commonly using chemical mutagens, ultraviolet irradiation and XL1-Red.

### MP6 enables direct RNA polymerase evolution during PACE

Next we compared the performance of MP1 and MP6 during PACE^10^,^11^,^31^,^32^,^33^ of T7 RNA polymerase. We previously showed that PACE can evolve T7 RNA polymerase to recognize the distant T3 promoter (P_T3_) (Supplementary Fig. 12), but only using either an intermediate evolutionary stepping stone (a T7/T3 hybrid promoter)^10^,^31^,^32^, or an initial period of evolutionary drift in the absence of selection pressure^11^. Without either an evolutionary stepping stone or initial evolutionary drift, PACE cannot evolve T7 RNAP variants that recognize P_T3_, leading to rapid phage washout^10^,^11^,^31^,^32^. We compared the ability of MP1 and MP6 with rapidly evolve P_T3_-active T7 RNAP variants in the absence of evolutionary drift, or under conditions in which the selection stringency was slightly reduced. Importantly, previous attempts of T7 RNAP evolution towards P_T3_ activity using MP1 required a drift period of virtually no selection pressure over 18 h to yield P_T3_-active variants, whereas the high or intermediate selection pressures resulted in rapid phage washout^11^.

**Figure 6:**
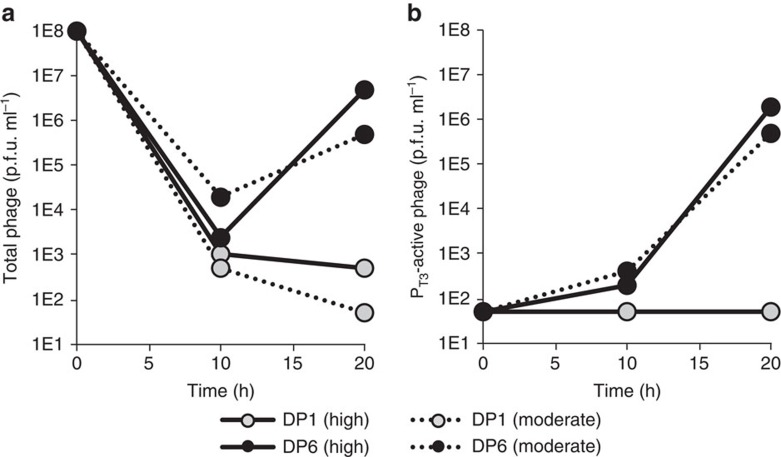
Continuous evolution of P_T3_-active RNAP variants. S1030 cells carrying an accessory plasmid (AP) encoding P_T3_ upstream of M13 bacteriophage *geneIII* and either DP1 or DP6 were infected with selection phage (SP) carrying wild-type T7 RNAP under conditions in which selection stringency was high (0 ng ml^−1^ ATc) or moderate (30 ng ml^−1^ ATc). (**a**) Total phage titres at 10 and 20 h after lagoon inoculation with the SP encoding T7 RNAP. (**b**) Titres of phage encoding evolved RNAP variants active on P_T3_. The limit of detection is 50 p.f.u. ml^−1^. p.f.u., plaque-forming units.

In agreement with our previous results, T7 RNAP phage added to lagoons fed by host cells harbouring MP1 under high or intermediate selection stringency rapidly washed out in <20 h of PACE when required to recognize P_T3_ (Fig. 6a). In contrast, both lagoons tested in which host cells harboured MP6 enabled the propagation of T7 RNAP phage on host cells requiring P_T3_ recognition under high or intermediate selection stringency, with nearly 10% of both populations after only 10 h of PACE exhibiting activity on P_T3_ by activity-dependent plaque assays (Fig. 6b). Sequencing confirmed mutations M219K/E222K/E222Q together with N748D in all surviving clones (Supplementary Fig. 13), in agreement with previous findings of our group and others^10^,^11^,^31^,^32^,^34^. The ability of MP6 to enable the discovery of P_T3_-active T7 RNAP variants highlights the ability of this MP to access mutations more efficiently, and thus mediate a more thorough and larger sampling of sequence space. Collectively, these results establish that the enhanced mutagenesis mediated by the new MPs can enable accelerated access to evolved proteins that are difficult to access directly using previous methods.

## Discussion

Using a systematic, mechanism-guided approach, we developed a series of vectors that express a variety of genes known to adversely affect DNA replication fidelity. In total we generated and assayed 80 candidate MPs with mutation rates spanning five orders of magnitude (Supplementary Figs 5 and 6 and Supplementary Tables 2 and 3). The resulting MPs enable highly potent, broad-spectrum, inducible, vector-based *in vivo* mutagenesis that rival the performance characteristics of popular *in vitro* methods such as error-prone PCR, while offering key advantages of *in vivo* mutagenesis. These advantages include enabling mutation and selection cycles to be coupled, and bypassing transformation efficiency bottlenecks that limit the size of populations that can be generated from DNA diversified *in vitro*. The MPs developed here offer major advantages over current *in vivo* mutagenesis methods such as chemical mutagens or base analogues, ultraviolet irradiation or constitutive hypermutator strains. Hypermutator strains, for example, generally suffer from poor transformation efficiency (XL1-Red, ∼1 × 10^6^ colony-forming units per μg plasmid DNA), high instability and narrow mutagenic spectra. MP6 increases the mutation rate of *E. coli* by 322,000-fold, and substantially exceeds both the mutation rate and the mutagenic spectra of XL1-Red. Importantly, MP6 can enable ∼2.3 substitutions per kbp in a gene of interest using phage vectors in a single generation, with additional increases in mutagenesis efficiency concomitant with longer propagation times.

To demonstrate the utility of these vectors, we used a whole-genome mutagenesis approach to evolve high-level antibiotic resistance in *E. coli*. In the absence of any prior selection or mutagenesis step, MP6 rapidly mediates the evolution of antibiotic resistance to many commonly used antibiotics within 18 h. The efficiency and effectiveness of antibiotic resistance mediated by MP6 compares favourably with that of a number of potent chemical mutagens (2AP, EMS and MNNG), ultravioet irradiation and the hypermutator strain XL1-Red. In addition, we observed that MP6 enables the continuous evolution of T7 RNA polymerase variants capable of initiating transcription at the non-cognate T3 promoter in <10 h, without requiring evolutionary stepping stones or an initial period of evolutionary drift. We anticipate that these MPs will broadly enable the use of *in vivo* mutagenesis to provide efficient access to rare solutions in sequence space that would otherwise be much more difficult to reach using current *in vitro* or *in vivo* methods. Importantly, the MPs offer non-targeted mutagenesis enhancements that complement sequence-targeted mutagenesis approaches such as MAGE^35^ or using CRISPR-Cas9 (ref. 36). Furthermore, the MPs operate largely independently of host recombination pathways and bias mutagenesis towards substitutions rather than insertions or deletions, both of which are desirable characteristics in library generation during directed evolution. The properties of MP6, which include very high mutagenesis efficiency, broad mutational spectrum, small-molecule inducibility and compatibility with a variety of bacterial strains, together represent a substantial advance in *in vivo* mutagenesis methodology for the laboratory evolution community.

## Methods

### General methods

All PCR reactions were performed using *Pfu*Turbo Cx polymerase (Agilent Technologies) or VeraSeq ULtra polymerase (Enzymatics). Water was purified using a MilliQ water purification system (Millipore, Billerica, MA). All MPs were constructed using USER cloning (New England Biolabs). Native *E. coli* genes were amplified by PCR directly from genomic DNA, and non-bacterial genes were synthesized as bacterial codon-optimized gBlocks Gene Fragments (Integrated DNA Technologies). All DNA cloning was carried out using NEB Turbo cells (New England Biolabs).

### General MP strain preparation

Mid-log-phase (OD_600_=∼0.5–0.8) cells of the strain of interest grown in 2xYT (United States Biological) were transformed with the desired MP, and recovered for 45 min in Davis rich media^11^ to suppress MP induction. All transformations were plated on 2xYT in 1.8% agar (United States Biological) containing 40 μg ml^−1^ chloramphenicol (Sigma Aldrich), 10 μg ml^−1^ fluconazole (TCI America), 10 μg ml^−1^ amphotericin B (TCI America) and 25 mM glucose (United States Biological) and grown for 12–18 h in a 37-°C incubator. Colonies transformed with the appropriate MP were picked the following day and grown in Davis rich media containing 40 μg ml^−1^ chloramphenicol, 10 μg ml^−1^ fluconazole and 10 μg ml^−1^ amphotericin B for 12–18 h. Following overnight growth of the MP-carrying strains, cultures were diluted 1,000-fold into fresh Davis rich media containing 40 μg ml^−1^ chloramphenicol, 10 μg ml^−1^ fluconazole and 10 μg ml^−1^ amphotericin B. The remainder of each experiment is described in each of the following sections.

### Rifampin-resistance assay

On reaching mid log-phase, cultures were induced with 25 mM arabinose (Davis rich media+arabinose) or suppressed with 25 mM glucose (Davis rich media only) and allowed to continue growth for an additional ∼18–24 h in a 37-°C shaker. The high arabinose concentration ensures sufficient induction of the plasmid-borne mutators MG1655 *ΔC shaker.* despite arabinose catabolism by this strain on glucose depletion. For XL1-Blue and XL1-Red strains, cultures were started directly from glycerol stocks according to the manufacturer's instructions and incubated for an identical amount of time as the MP-carrying strains. After overnight growth, cultures were serially diluted in 10-fold increments and plated on 2xYT agar containing 10 μg ml^−1^ fluconazole, 10 μg ml^−1^ amphotericin B and 100 mM glucose±100 μg ml^−1^ rifampin. After 18–24 h, the number of colonies on the glucose±rifampin plates was counted for each culture. The mutation efficiency induced by the MP (*t*_bp_, substitutions per bp per generation) was calculated using the equation: *μ*_bp_*=f/*[*R* × ln(*N/N*_0_)], where *f* is the frequency of rifampin-resistant mutants (as compared with the glucose control), *R* is the number of unique sites yielding rifampin resistance (77 previously identified sites; we identified only 21 sites across both *rpoB* clusters in our experiments), *N* is the final population size and *N*_*0*_ is the population size at which resistance is first observed (empirically determined to be ∼1.5 × 10^7^). To calculate *μ*_G_, *μ*_bp_ was multiplied by the genome size, which for MG1655 was 4.64 × 10^6^ bp.

### Episomal *lacZ* reversion assay

On reaching mid log-phase, the cultures were induced with 25 mM arabinose or suppressed with 25 mM glucose, and allowed to continue growth for an additional ∼18–24 h. After overnight growth, the cultures were centrifuged for 2 min at 10,000 × relative centrifugal force (r.c.f.) and resuspended in an equal volume of 10% glycerol. This procedure was carried out twice to remove trace glucose or other carbon sources from the supernatant before plating. Washed cells were serially diluted in 10-fold increments using 10% glycerol and plated on M9 minimal media agar supplemented with 5 mM MgSO_4_, 0.01% thiamine, 335 phase, the cultures were induced with 25 mM arabinose or suppressed with 25 mM glucose, and allowed to continue growth for an additional ∼18–24 h. After overnight *lacZ* reversion, and not purely due to extracellular lactose hydrolysis. After extended growth (∼24–36 h), the fraction of lactose-catabolizing colonies was calculated using the number of blue colonies on the lactose plates versus the total number of colonies on the glucose plates.

### Phage *lacZ* inactivation assay

On reaching mid log-phase, the cultures were induced with 25 mM arabinose or suppressed with 25 mM glucose, allowed to grow for an additional 0–2 h, then, in the case of strain S1030, infected with SP063 phage, and allowed to grow for an additional ∼18–24 h. For S1021 and XL1-Red (Agilent Technologies), SP063 DNA was miniprepped from infected S1030 cells and electroporated into these strains as they both lack *F′* episomes. For *F*^−^ cells, cultures were either induced for 2 h before being made electrocompotent, induced immediately following transformation, induced both before and following electroporation, or not induced at all. After overnight growth and phage propagation, the cultures were centrifuged for 2 min at 10,000 × r.c.f. and the supernatant was filtered through a 0.2-μm PVDF filter (Millipore). The supernatant was serially diluted in 10-fold increments using Davis rich media and plaqued on S1030 cells using 1.8% 2xYT agar for the bottom layer and 0.6% 2xYT agar supplemented with 400 μg ml^−1^ Bluo-Gal (Life Technologies) for the top layer. The fraction of white or light blue plaques (lacZ^−^ phenotype) was counted as a function of all plaques (blue+light blue+white), and used as a measure of mutation frequency for the *lacZ* cassette.

### Sanger sequencing of *rpoB* mutations

Rifampin-resistant colonies were picked into 96-well plates and grown overnight in Davis rich media supplemented with 100 μg ml^−1^ rifampin. Following overnight growth, 10-μl aliquots were heated at 100 °C for 10 min, followed by PCR using primers AB1678 (5′- AATGTCAAATCCGTGGCGTGAC ) and AB1682 (5′- TTCACCCGGATACATCTCGTCTTC ) to amplify an *rpoB* fragment containing both clusters I and II. Each fragment was sequenced twice using primers AB1680 (5′- CGGAAGGCACCGTAAAAGACAT ) and AB1683 (5′- CGTGTAGAGCGTGCGGTGAAA ).

### High-throughput sequencing of *lacZ* mutations

SP063 phage that was propagated using S1030 carrying MP1, MP4 or MP6, produced by XL1-Red following SP063 DNA electroporation, or the unmutated stock phage was amplified by PCR using primers AB437 (5′- GGCGCTGGTAAACCATATG ) and DB213 (5′- GGAAACCGAGGAAACGCAA ) to yield an ∼3,400-bp fragment containing the *lacZ* gene. SP063 phage that was propagated under similar conditions on S1030 cells was used as the negative control. Three biological replicates were carried out for each of the aforementioned samples. The resulting PCR products were purified by gel electrophoresis using a 1% agarose gel and prepared for HTS using a Nextera kit (Illumina) and a previously described procedure^33^. Briefly, 4 μl of DNA (2.5 ng μl^−1^), 5 μl TD buffer and 1 μl TDE1 were mixed together and then heated to 55 °C for 5 min. After purification (Zymo DNA purification kit), the resultant ‘tagmented' DNA samples were amplified with Illumina-supplied primers using the manufacturer's protocol. The resulting PCR products were then purified using AMPure XP beads and the final concentration of DNA was quantified using PicoGreen (Invitrogen) and qPCR. The samples were sequenced on a MiSeq Sequencer (Illumina) in 2 × 300 paired-end runs using the manufacturer's reagents following the manufacturer's protocols.

### High-throughput sequencing data analysis

A previously described custom MATLAB script^33^ (available on request) was used to align MiSeq reads with *Q* score ⩾30 to the wild-type sequence and count the nucleotide positions from which the experimental sample deviates from the wild-type sequence, yielding called mutations with ⩾99.9% accuracy, corresponding to >3 s.d. above the mean error rate of the MiSeq high-throughput sequencing reads. To compensate for systemic sample preparation and sequencing errors, the observed fraction of mutations at each nucleotide position of the wild-type *lacZ* reference gene was subtracted from the fraction of mutations in a given experimental sample to result in the ‘corrected fraction mutated'. Mutations were defined as nucleotide positions with a corrected fraction mutation that is both greater than the average corrected fraction mutated of the treatment of interest and at least one s.d. higher than the corrected fraction mutation of the wild-type reference sequence. Duplicates belonging to set of paired-end reads were treated as a single sample, while duplicate reads of the same region with alternative adaptor/index sequences were not removed so as not to introduce bias into the sequencing analysis. This process yielded an average of ∼50,000 reads per position for each of the sequenced samples.

### Evolution of novel antibiotic resistance

MG1655 Δ*recA::apra* cells without an MP or carrying MP1, MP4 or MP6 were grown for 18–21 h in Davis rich media containing 40 μg ml^−1^ chloramphenicol, 10 μg ml^−1^ fluconazole, 10 μg ml^−1^ Amphotericin B and supplemented with 200 mM arabinose to induce the MPs. Small molecule and ultraviolet mutagenesis was carried out as previously described^30^. For 2AP treatment, log-phase MG1655 Δ*recA::apra* cells were diluted to ∼1,000 cells, the media was supplemented with 700 μg ml^−1^ 2AP (TCI America), and the culture was allowed for grow at 37 °C for an additional 18–21 h. For EMS treatment, 2 ml of a log-phase MG1655 Δ*recA::apra* culture (∼1 × 10^8^–1 × 10^9^ cells) was centrifuged, washed twice with 1 ml A buffer on ice, then supplemented with 14 μl EMS (TCI America). Cells were lightly vortexed, and allowed to shake at 200 r.p.m. at 37 °C for 45 min. After this time, the culture was centrifuged, washed twice with 1 ml A buffer on ice, diluted by 20-fold into Davis rich media without antibiotics, and allowed to grow for 18–21 h. For MNNG treatment, 2 ml of a log-phase MG1655 Δ*recA::apra* culture (∼1 × 10^8^–1 × 10^9^ cells) was centrifuged, washed twice with 1 ml citrate buffer (pH 5.5) on ice, supplemented with 111 μl of 1 mg ml^−1^ MNNG (TCI America) and placed in a 37 °C water bath for 30 min. Following treatment, the cells were centrifuged, washed twice with 1 ml 0.1 M potassium phosphate buffer (pH 7.0), diluted by fourfold into Davis rich media without antibiotics, and allowed to grow for 18–21 h. For ultraviolet irradiation, 2 ml of a log-phase MG1655 Δ*recA::apra* culture (∼1 × 10^8^–1 × 10^9^ cells) was centrifuged, resuspended in 1 ml 0.1 M MgSO4 and placed on ice for 10 min. Cells were placed in a Petri dish and exposed to ultraviolet light from a SM-36-2GR ultraviolet lamp (American Air & Water) for 1 min, uncovered, at a distance of ∼10 cm. Immediately following ultraviolet exposure, cells were diluted by 20-fold into Davis rich media without antibiotics, and allowed to grow for 18–21 h. For XL1-Blue and XL1-Red strains, cultures were started directly from glycerol stocks according to the manufacturer's instructions and allowed to grow for 18–21 h in Davis rich media. Following overnight growth, all cultures were serially diluted in Davis rich media and plated on 2xYT agar containing 10 μg ml^−1^ fluconazole and 10 μg ml^−1^ amphotericin B 100 mM glucose±the appropriate antibiotic. After overnight growth (∼18–24 h), the numbers of colonies on the glucose±antibiotics plates were counted.

### Continuous evolution of P_T3_-active T7 RNAP variants

Two modified versions of MP1 and MP6 (DP1 and DP6, respectively) were generated to enable robust phage propagation during PACE. These MPs carry all of the components of their respective MPs, in addition to the previously described anhydrotetracycline (ATc)-dependent drift promoter driving *geneIII* (ref. 11). S1030 strains carrying either MP in addition to the P_T3_ accessory plasmid were inoculated into host-cell cultures (chemostats) and grown at a dilution rate of 1.6 vol h^−1^ as previously described^11^. Lagoons flowing from the respective chemostats were maintained at 40 ml, diluted at 0.75 vol h^−1^, and supplemented with either 25 mM arabinose only (high stringency) or 25 mM arabinose with 30 ng ml^−1^ ATc (intermediate stringency) for 8 h before infection with packaged T7 RNAP selection phage. We note that concentrations exceeding 30 ng ml^−1^ for extended timeframes during PACE (>24 h) result in excision of the evolving gene from the selection phage. As continuous flow conditions effectively enrich for selection phages capable of rapid replication, selection phage with smaller genomes are rapidly enriched to totally dominate the evolving pool. Each lagoon was infected with 4 × 10^9^ plaque-forming unit (p.f.u.), resulting in an initial titer of 10^8^ p.f.u. ml^−1^ of the lagoon. Samples were taken 10 and 20 h after infection, centrifuged at 10,000 r.c.f. for 2 min, then sterile filtered with a 0.2-μm filter and stored overnight at 4 °C. Phage aliquots were titered on S1030 cells carrying either the PSP-*geneIII* accessory plasmid (total phage) or the P_T3_-*geneIII* AP (P_T3_-active phage).

## Additional information

**Accession codes:** The high-throughput sequencing data has been deposited in the NCBI Sequence Read Archive database under Bioproject accession code PRJNA294841.

**How to cite this article:** Badran, A. H. & Liu, D. R. Development of potent *in vivo* mutagenesis plasmids with broad mutational spectra. *Nat. Commun.* 6:8425 doi: 10.1038/ncomms9425 (2015).

## Supplementary Material

Supplementary Information Supplementary Figures 1-13, Supplementary Tables 1-6, Supplementary Notes and Supplementary References
