# Supplementary material for: Development of potent in vivo mutagenesis plasmids with broad mutational spectra
Source: Nat Commun. 2015 Oct 7;6:8425. doi: 10.1038/ncomms9425 (PMC4633624; doi:10.1038/ncomms9425)
Supplement: Supplementary Information — Supplementary Figures 1-13, Supplementary Tables 1-6, Supplementary Notes and Supplementary References [file ncomms9425-s1.pdf]

Supplementary Figures

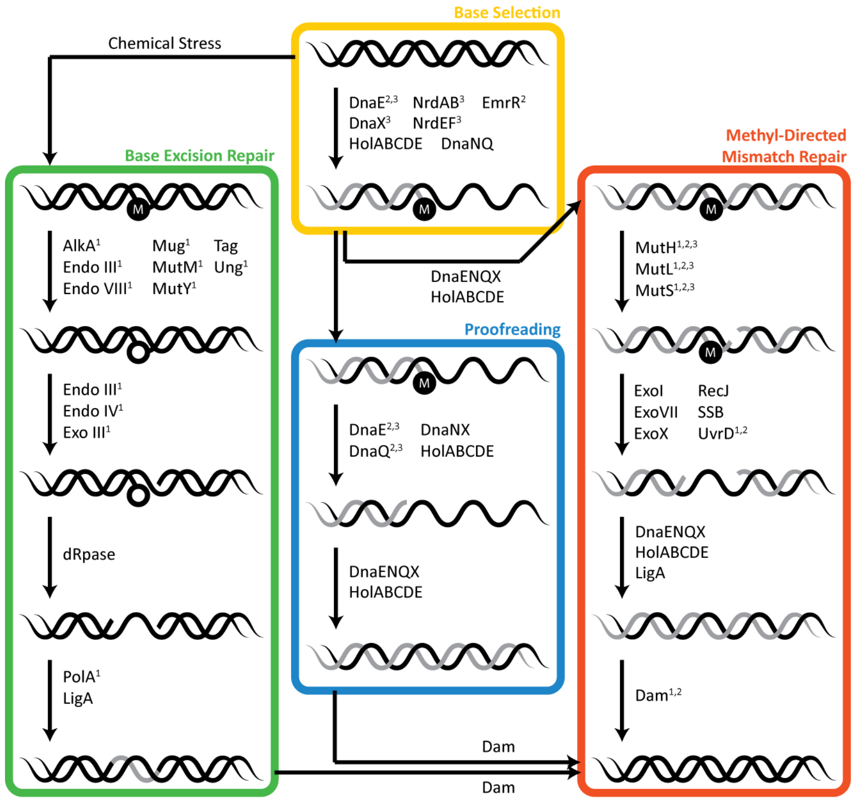

Supplementary Figure 1. Summary of major pathways that influence *E. coli* DNA replication fidelity. Steps during replication and mutation correction are grouped according to their mechanisms of action. Methylated DNA is shown in black, unmethylated DNA is shown in grey, and the mutation to be corrected is depicted as "M". Gene superscripts denote if a mutator phenotype results upon gene deletion[1] (1), gene overexpression[2-6] (2) or modification of the chromosomal allele to circumvent potential knockout lethality[2-7] (3).

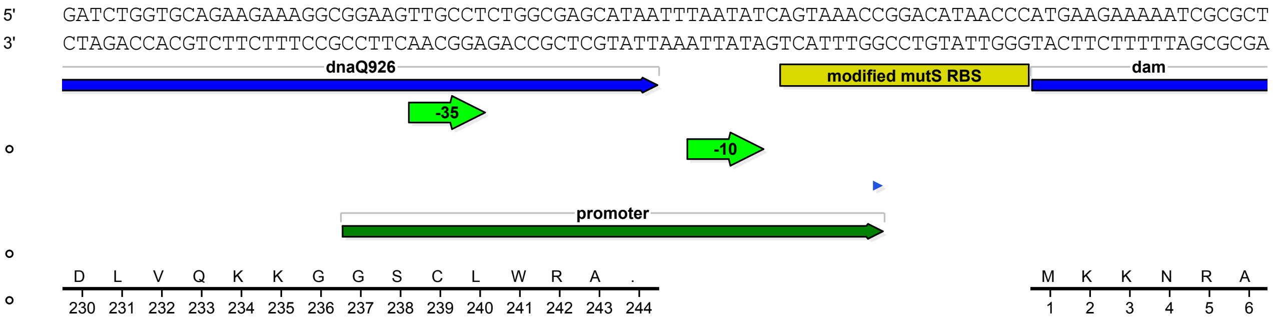

Supplementary Figure 2. Cryptic σ^{70} promoter at the 3' end of the *dnaQ926* ORF. Annotated sequence of the predicted σ^{70} promoter in MP3 bridging the 3' end of the *dnaQ926* ORF and RBS driving the *dam* ORF.

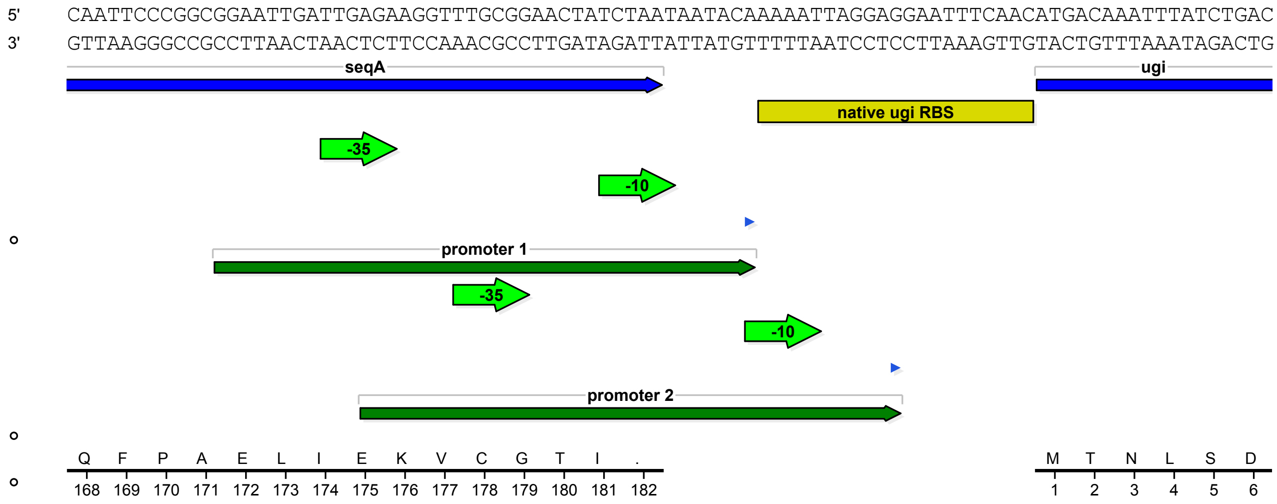

Supplementary Figure 3. Cryptic σ^{70} promoters at the 3' end of the *seqA* ORF. Annotated sequence of the predicted σ^{70} promoters in MP5 bridging the 3' end of the *seqA* ORF and native RBS driving the *ugi* ORF.

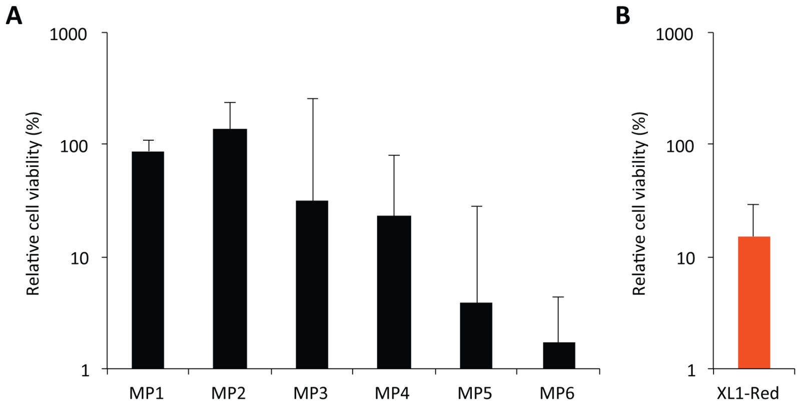

Supplementary Figure 4. Effect of MPs on host viability under induced conditions. A) Relative cell viability was calculated as the fractional cell titer following arabinose induction as compared to the uninduced control for each MP. Viability is anti-correlated with mutagenic potency at high levels. B) The XL1-Red strain shows the expected level of viability (compared to the control, XL1-Blue) given its mutagenic potency as compared to the designed MPs.

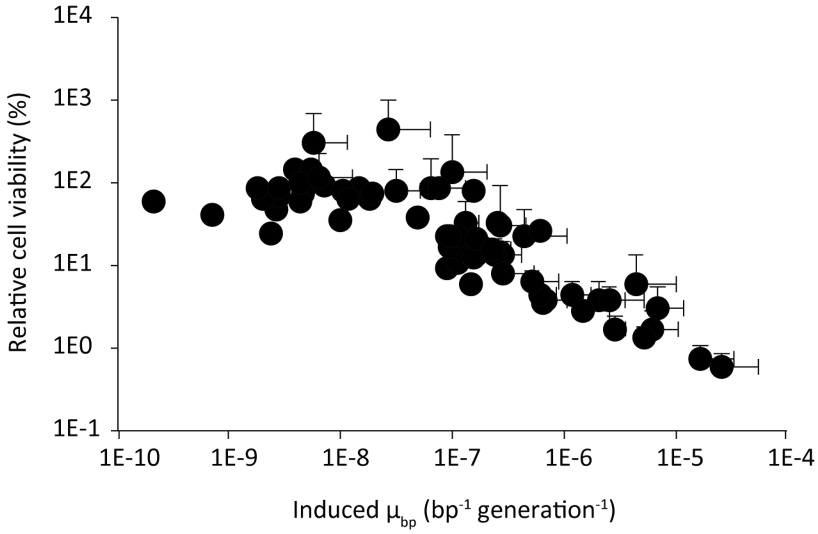

Supplementary Figure 5. Relationship between host viability and induced levels of mutagenesis for all MPs. Low potency MPs which induce up to ~4x10^{-7} substitutions/bp/generation were well tolerated by the *E. coli* MG1655 Δ*recA::apra* host, while higher levels of mutagenesis generally resulted in a reduced host viability, as expected. This inflection point corresponds to ~1.9 substitutions/genome/generation for wild-type *E. coli MG1566* (genome size = 4.64 x 10^6 bp).

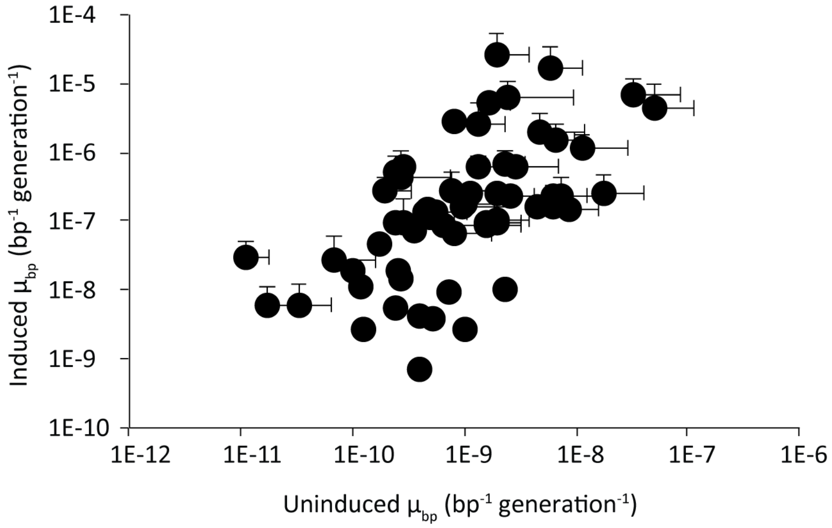

Supplementary Figure 6. Relationship between uninduced and induced levels of mutagenesis for all MPs. Higher levels of background (uninduced mutagenesis) were generally accompanied by an increase in overall MP mutagenesis upon induction. The full data set is provided in Supplementary Table 2.

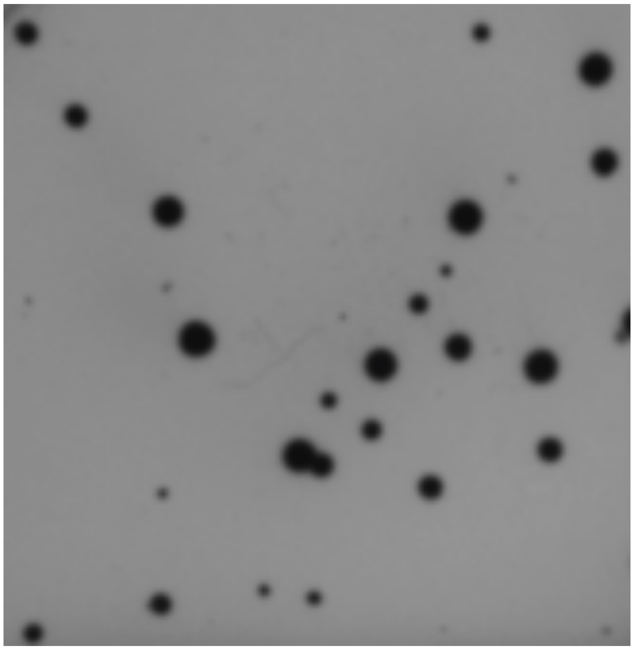

Supplementary Figure 7. Plaque assay of the *lacZ*-carrying M13 phage SP063. SP063 carries the wild-type E. coli β-galactosidase gene with a consensus ribosome-binding site directly downstream of *geneIII*. Plating using soft agar containing S1030 cells in the presence of the X-Gal analog Bluo-Gal (Life Technologies) results in a strong, deep blue plaques (shown here as dark circles).

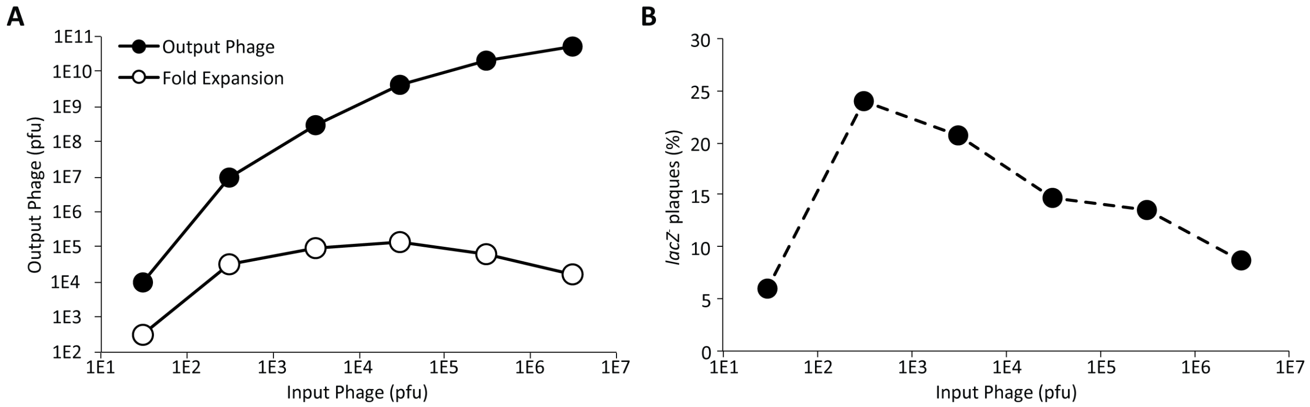

Supplementary Figure 8. Optimization of phage inoculant for optimal expansion and mutagenesis. A) *E. coli* S1030 carrying MP6 were induced with arabinose during log-phase growth and concomitantly infected with serially diluted SP063 phage. Phage were titered after overnight propagation and the fold expansion of the phage population was determined. B) The percentage of white and light blue (lacZ$^-$) plaques in the presence of Bluo-Gal suggests a correlation between phage population expansion and mutagenesis efficiency.

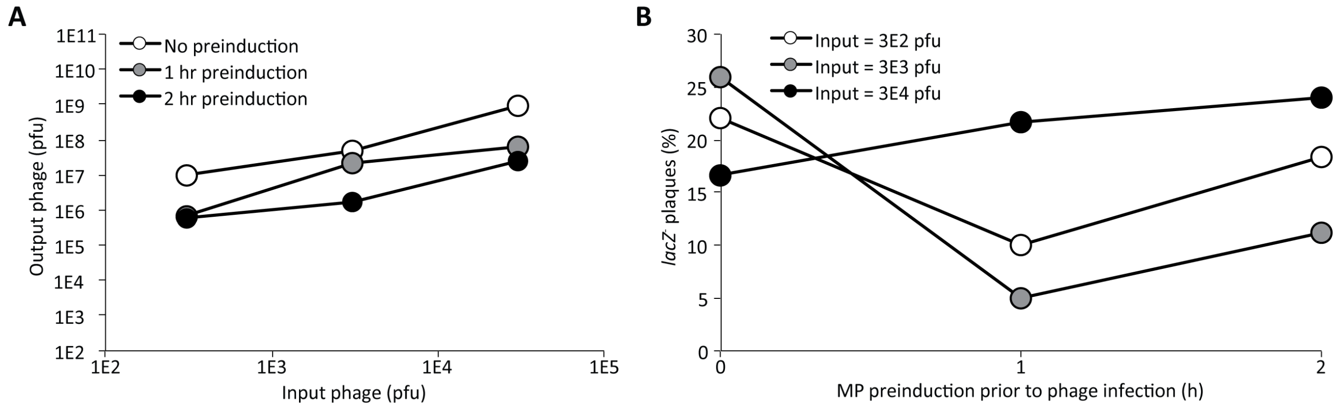

Supplementary Figure 9. Effect of MP pre-induction on phage mutagenesis. *E. coli* S1030 carrying MP6 were induced with arabinose during log-phase growth and concomitantly infected with SP063 phage at defined titers. Alternatively, infection was delayed for 1 h or 2 h. Phage were titered after overnight propagation and the percentage of white plaques in the presence of Bluo-Gal was counted.

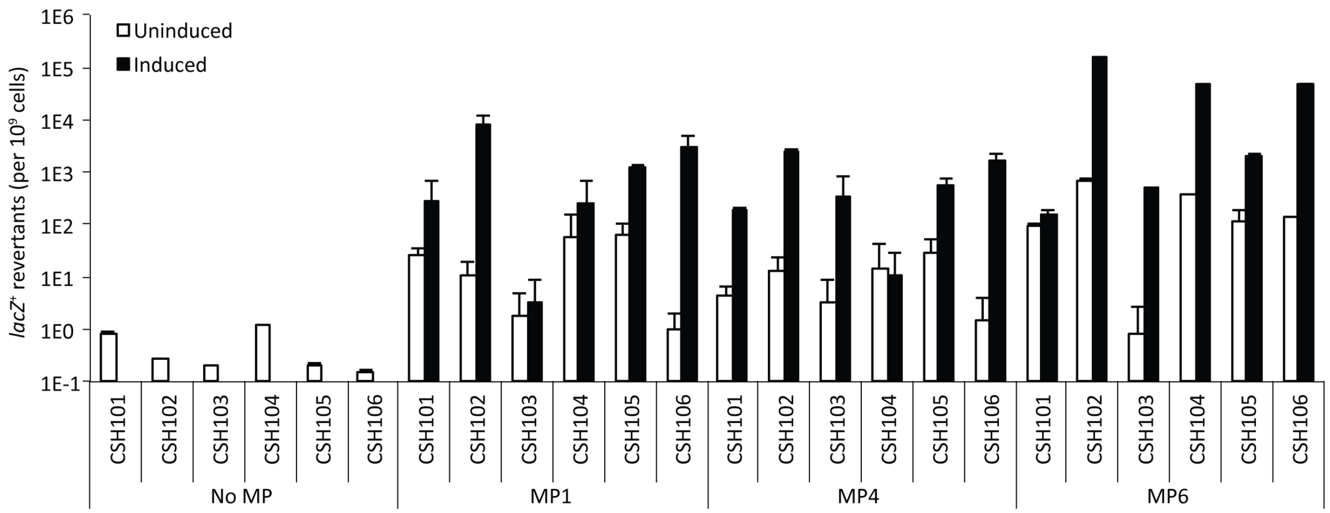

Supplementary Figure 10. Analysis of *F'* episomal mutations rates using various MPs. The frequency of *lacZ*+ revertants is the fraction of colonies surviving on lactose as the sole carbon source as compared to the total colony count (colonies that survive on glucose as sole carbon source). Each strain reports the MP's ability to increase the frequency of a specific mutation type.

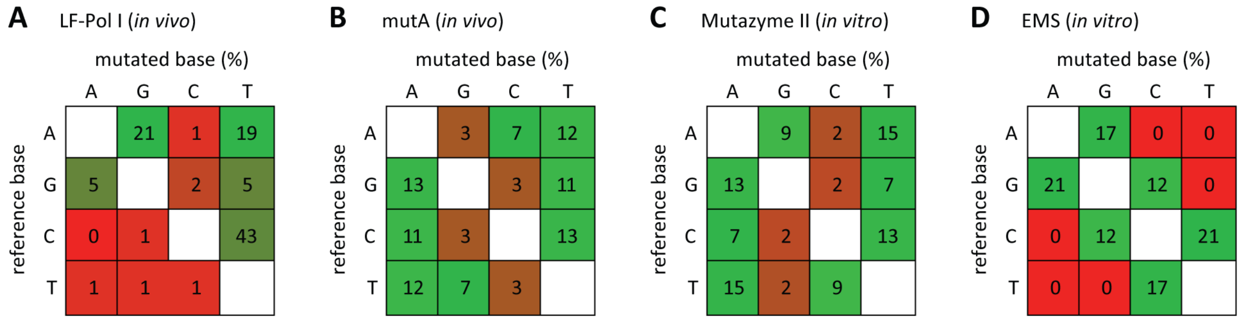

Supplementary Figure 11. Mutagenic spectra of commonly used mutagenesis techniques. (A-D) Previously reported mutagenic spectra of four commonly used mutagenesis methods: LF Pol I[8], *mutA*[9], Mutazyme II[10] and EMS[11].

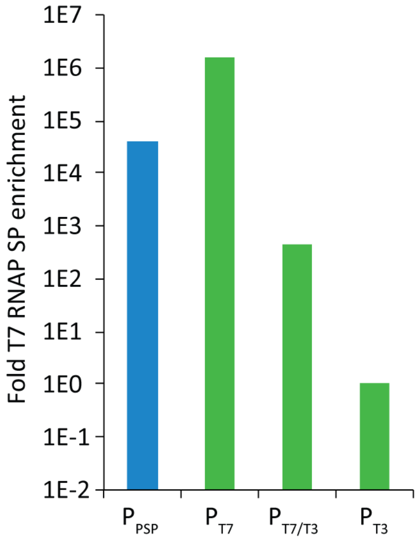

Supplementary Figure 12. Activity of T7 RNAP on cognate and non-cognate promoters. Log-phase S1030 cells carrying accessory plasmids (APs) with a *geneIII* cassette with an upstream phage shock protein (PSP), T7, hybrid T7/T3, or T3 promoter were infected with selection phage (SPs) carrying the wild-type T7 RNAP. The fraction of output phage vs. input phage indirectly reports on the activity of the T7 RNAP on the various promoters. Enrichment factors of ~100 or less indicate extremely weak to non-existent activity.

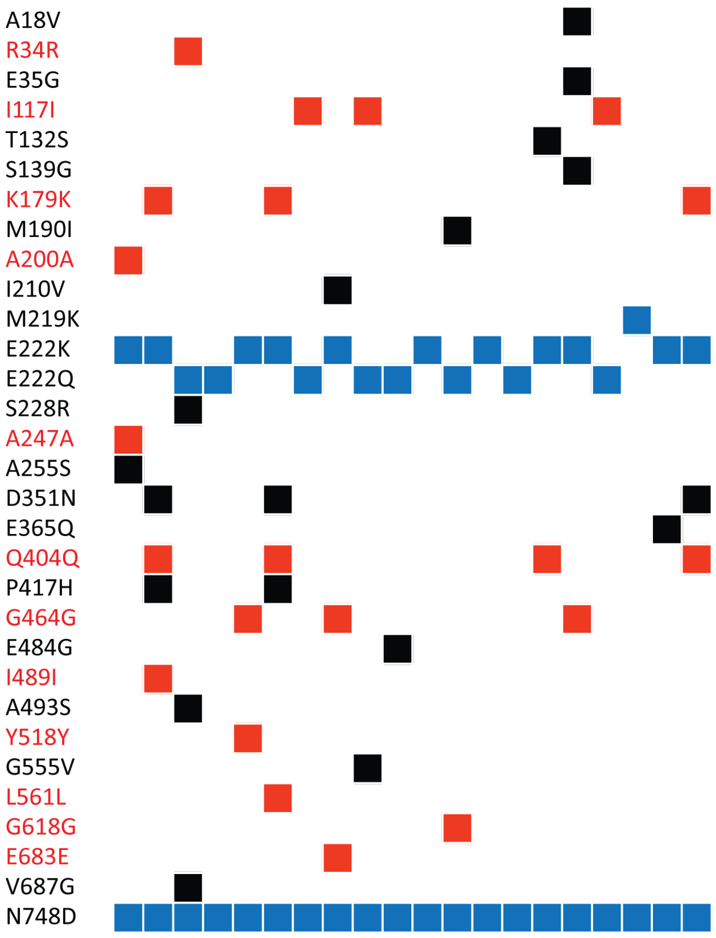

Supplementary Figure 13. Single-phage plaque sequencing of P_{T3}-active SPs. Single phage plaques at 10 h of PACE using the P_{T3} AP were isolated and subjected to Sanger sequencing. All clones carried T7 RNAP variants with conserved mutations known to confer activity on P_{T3} (blue), as well as additional mutations that may further enhance activity (black). Silent mutations were also detected (red).

Supplementary Tables

Strain	CGSC #	Genotype
MG1655 Δ*recA*	12492	*F⁻ ΔrecA1918::apra, rph-1 λ⁻*
CSH101	8095	*F' lacI373 lacZ571 / ara-600 Δ(gpt-lac)5 relA1 spoT1 thiE1 λ⁻*
CSH102	8096	*F' lacI373 lacZ572 / ara-600 Δ(gpt-lac)5 relA1 spoT1 thiE1 λ⁻*
CSH103	8097	*F' lacI373 lacZ573 / ara-600 Δ(gpt-lac)5 relA1 spoT1 thiE1 λ⁻*
CSH104	8098	*F' lacI373 lacZ574 / ara-600 Δ(gpt-lac)5 relA1 spoT1 thiE1 λ⁻*
CSH105	8099	*F' lacI373 lacZ575 / ara-600 Δ(gpt-lac)5 relA1 spoT1 thiE1 λ⁻*
CSH106	8100	*F' lacI373 lacZ576 / ara-600 Δ(gpt-lac)5 relA1 spoT1 thiE1 λ⁻*
S1030	N/A	*F' proA+B+ Δ(lacIZY) zzf::Tn10* $lacI^{Q1}$ P_{N25}*-tetR luxCDE / endA1 recA1 galE15 galK16 nupG rpsL ΔlacIZYA araD139 Δ(ara,leu)7697 mcrA Δ(mrr-hsdRMS-mcrBC) proBA::pir116 araE201 ΔrpoZ Δflu ΔcsgABCDEFG ΔpgaC λ⁻*
S1021	N/A	*F⁻ endA1 recA1 galE15 galK16 nupG rpsL ΔlacIZYA araD139 Δ(ara,leu)7697 mcrA Δ(mrr-hsdRMS-mcrBC) proBA::pir116 araE201 ΔrpoZ Δflu ΔcsgABCDEFG ΔpgaC λ⁻*

Supplementary Table 1. Summary of all strains used in this study. Strains that were requested from the Yale Coli Genetic Stock Center (CGSC) show the corresponding strain numbers.

Name	Genes encoded on the MP (native order)				
**MP1**	dnaQ926[12]	umuD'	umuC	recA730[13]	
**MP2**	dnaQ926				
MP-B2	dnaE74[16]				
MP-B4	dnaE486[4]				
MP-B5	dnaE1026[17]				
MP-C2	dnaX36[19]				
MP-C3	dnaX2016[19]				
MP-D3	dnaQ926	dnaE486			
MP-D4	dnaQ926	dnaE1026			
MP-E	dnaQ926	dnaX36			
MP-E2	dnaQ926	dnaX2016			
MP-F2	dnaQ926	mutS538[6]			
MP-F3	dnaQ926	mutS503[6]			
MP-H	dnaQ926	mutL705[2]			
MP-H2	dnaQ926	mutL713[2]			
MP-H3	dnaQ926	mutL(R261H)[3]			
MP-H4	dnaQ926	mutL(K307A)[3]			
MP-I	dnaQ926	mutH(E56A)[3]			
MP-I2	dnaQ926	mutH(K79E)[3]			
MP-I3	dnaQ926	mutH(K116E)[3]			
MP-J	rpsD12[21]				
MP-J2	rpsD14[21]				
MP-J3	rpsD16[21]				
**MP3**	dnaQ926	dam[22]			
MP-K7	dnaQ926	dam	emrR[5]		
**MP4**	dnaQ926	dam	seqA[5]		
MP-K9	dnaQ926	dam	mutSΔN[5]		
MP-K10	dnaQ926	dam	seqA	emrR	
MP-K11	dnaQ926	dam	seqA	mutSΔN	
MP-K12	dnaQ926	dam	seqA	dinB[16]	
MP-K13	dnaQ926	dam	seqA	polB[26]	
MP-K14	dnaQ926	dam	**seqA***		
MP-L	polB				
MP-L2	polB(D156A)[26]				
MP-P	dnaQ926	dam	seqA	emrR	mutH(E56A)
MP-P3	dnaQ926	dam	seqA	emrR	mutL713
MP-P4	dnaQ926	dam	seqA	emrR	mutS503
MP-P5	dnaQ926	dam	seqA	emrR	mutSΔN
MP-P6	dnaQ926	dam	seqA	emrR	dinB
MP-P7	dnaQ926	dam	seqA	emrR	polB

Name	Genes encoded on the MP (native order)						
MP-P8	dnaQ926	dam	seqA	emrR	ugi[14]	AID[15]	
MP-P9	dnaQ926	dam	seqA	emrR	ugi	APOBEC1[15]	
**MP6**	dnaQ926	dam	seqA	emrR	ugi	CDA1[15]	
MP-P11	dnaQ926	dam	seqA	emrR	ugi	CDA1	mutSΔN
MP-Q	dnaQ926	dam	seqA	rsmE[18]			
MP-Q2	dnaQ926	dam	seqA	cchA[18]			
MP-Q3	dnaQ926	dam	seqA	yffI[18]			
MP-Q4	dnaQ926	dam	seqA	yfjY[5]			
MP-Q5	dnaQ926	dam	seqA	ugi	AID		
MP-Q6	dnaQ926	dam	seqA	ugi	APOBEC1		
**MP5**	dnaQ926	dam	seqA	ugi	CDA1		
MP-Q8	dnaQ926	dam	seqA	nrdAB[7]			
MP-Q9	dnaQ926	dam	seqA	nrdA(H59A)B[7]			
MP-Q10	dnaQ926	dam	seqA	nrdA(A65V)B[20]			
MP-Q11	dnaQ926	dam	seqA	nrdA(A301V)B[20]			
MP-Q12	dnaQ926	dam	seqA	nrdAB(P334L)[20]			
MP-Q13	dnaQ926	dam	seqA	nrdEF[7]			
MP-R	dnaQ926	dam	seqA	ugi	AID (opt)		
MP-R2	dnaQ926	dam	seqA	ugi	APOBEC1 (opt)		
MP-R3	dnaQ926	dam	seqA	ugi	CDA1 (opt)		
MP-R4	dnaQ926	dam	seqA	emrR	ugi	AID (opt)	
MP-R5	dnaQ926	dam	seqA	emrR	ugi	APOBEC1 (opt)	
MP-R6	dnaQ926	dam	seqA	emrR	ugi	CDA1 (opt)	
MP-S	dnaQ926	MAG1[23]					
MP-S2	dnaQ926	AAG(Y127I-H136L)[24]					
MP-S3	dnaQ926	Δ80-AAG(Y127I-H136L)[24]					
MP-T	dnaQ926	dam	seqA	emrR	ugi	AID(7)[25]	
MP-T2	dnaQ926	dam	seqA	emrR	ugi	AID(7.3)[25]	
MP-T3	dnaQ926	dam	seqA	emrR	ugi	AID(7.3.5)[25]	
MP-T4	dnaQ926	dam	seqA	emrR	ugi	AID(7.3.3)[25]	
MP-T5	dnaQ926	dam	seqA	emrR	ugi	AID(7.3.1)[25]	
MP-T6	dnaQ926	dam	seqA	emrR	ugi	AID(7.3.2)[25]	
MP-U	**dnaQ926***	dam	seqA	emrR	ugi	CDA1	
MP-U2	dnaQ926	**dam***	seqA	emrR	ugi	CDA1	
MP-U3	dnaQ926	dam	seqA	**emrR***	ugi	CDA1	
MP-U4	dnaQ926	dam	seqA	emrR	ugi	**CDA1***	
MP-V	BR13[27]	dam	seqA	emrR	ugi	CDA1	
MP-V2	BRM1[27]	dam	seqA	emrR	ugi	CDA1	
MP-V3	BR11[27]	dam	seqA	emrR	ugi	CDA1	
MP-V4	BR6[27]	dam	seqA	emrR	ugi	CDA1	
MP-V5	BR1[27]	dam	seqA	emrR	ugi	CDA1	

Supplementary Table 2. Summary of all ORFs carried by the MPs. All MPs use the identical vector backbone: a cloDF13 origin of replication (20-40 copies/cell), a chloramphenicol resistance cassette, the arabinose responsive promoter P_{BAD} driving the mutator genes, and the weak promoter P_C driving *araC*. Genes carried by each MP are arranged in the order found in the table and are highlighted according to their mechanism of action and/or the canonical repair pathway that they disrupt: proofreading (blue), translesion synthesis (purple), methyl-directed mismatch repair (red), base excision repair (green), base selection (yellow) and unknown (black). Additional optimizations included: codon usage optimization (opt) and increased ribosome-binding site strength (*). Boxes are not drawn to scale.

Name	Uninduced μ_{bp}	Induced μ_{bp}	Viability (%)
None	3.26E-11	6.07E-12	100
**MP1**	8.20E-10	6.42E-08	87.5
**MP2**	2.77E-10	9.85E-08	136.8
MP-B2	0.00E+00	0.00E+00	25.4
MP-B4	0.00E+00	0.00E+00	45.8
MP-B5	0.00E+00	0.00E+00	55.9
MP-C2	0.00E+00	0.00E+00	63.6
MP-C3	0.00E+00	0.00E+00	53.4
MP-D3	0.00E+00	2.31E-09	24.2
MP-D4	0.00E+00	2.59E-09	68.6
MP-E	0.00E+00	2.13E-10	61
MP-E2	3.98E-10	6.95E-10	40.7
MP-F2	9.83E-10	2.68E-09	48.3
MP-F3	0.00E+00	4.59E-09	73.7
MP-H	0.00E+00	7.07E-09	94.1
MP-H2	7.15E-10	9.61E-09	35.6
MP-H3	1.27E-10	2.75E-09	71.2
MP-H4	0.00E+00	2.03E-09	63.6
MP-I	0.00E+00	4.26E-09	61
MP-I2	0.00E+00	1.75E-09	83.9
MP-I3	0.00E+00	2.72E-09	89
MP-J	0.00E+00	0.00E+00	223.7
MP-J2	0.00E+00	0.00E+00	142.4
MP-J3	0.00E+00	0.00E+00	137.3
**MP3**	1.74E-08	2.66E-07	31.2
MP-K7	6.53E-10	8.99E-08	9.4
**MP4**	2.73E-10	4.38E-07	22.9
MP-K9	1.55E-09	8.99E-08	23.4
MP-K10	1.12E-11	3.07E-08	80.6
MP-K11	9.87E-11	1.85E-08	63.6
MP-K12	2.68E-10	1.48E-08	86.4
MP-K13	0.00E+00	1.83E-08	63.6
MP-K14	1.12E-09	0.00E+00	0.8
MP-L	0.00E+00	0.00E+00	129.7
MP-L2	0.00E+00	0.00E+00	93.8
MP-P	5.32E-10	3.84E-09	144.9
MP-P3	1.14E-09	2.51E-07	33.3
MP-P4	4.58E-09	1.57E-07	81.4
MP-P5	2.83E-10	6.06E-07	26.7
MP-P6	4.90E-10	1.13E-07	10.7
MP-P7	3.54E-10	7.58E-08	83.9
MP-P8	5.49E-10	1.31E-07	32.8
MP-P9	3.28E-08	7.04E-06	2.9
**MP6**	2.44E-09	6.24E-06	1.7
MP-P11	1.66E-09	5.30E-06	1.4
MP-Q	4.49E-10	1.35E-07	14.7
MP-Q2	1.55E-09	9.24E-08	20.6
MP-Q3	2.37E-10	9.36E-08	16.8
MP-Q4	1.94E-09	2.64E-07	15
MP-Q5	1.92E-09	1.09E-07	22.5
MP-Q6	9.86E-10	1.83E-07	16.3
**MP5**	4.79E-09	2.01E-06	3.8
MP-Q8	1.70E-10	4.82E-08	37.4
MP-Q9	2.50E-10	1.88E-08	73.7
MP-Q10	2.26E-09	1.04E-08	81.4
MP-Q11	1.16E-10	1.15E-08	66.1
MP-Q12	2.40E-10	5.48E-09	144.9
MP-Q13	3.98E-10	4.24E-09	101.7
MP-R	9.47E-10	1.64E-07	13.9
MP-R2	7.52E-09	2.37E-07	13.1
MP-R3	1.12E-08	1.17E-06	4.3
MP-R4	2.34E-09	6.94E-07	3.7
MP-R5	4.90E-08	4.35E-06	5.8
MP-R6	5.80E-09	1.71E-05	0.7
MP-S	3.23E-11	6.29E-09	115.3
MP-S2	1.73E-11	5.76E-09	313
MP-S3	6.85E-11	2.68E-08	428.4
MP-T	1.92E-10	2.77E-07	13.6
MP-T2	1.93E-09	9.59E-08	23.1
MP-T3	8.70E-09	1.55E-07	12.6
MP-T4	6.09E-09	2.42E-07	13.7
MP-T5	6.28E-09	1.65E-07	21.7
MP-T6	2.50E-09	2.29E-07	16
MP-U	1.90E-09	2.66E-05	0.6
MP-U2	8.02E-10	2.88E-06	1.7
MP-U3	6.66E-09	1.49E-06	2.8
MP-U4	1.36E-09	2.51E-06	3.7
MP-V	4.59E-10	1.49E-07	5.7
MP-V2	7.79E-10	2.88E-07	7.9
MP-V3	2.95E-09	6.44E-07	3.4
MP-V4	1.35E-09	5.98E-07	4.6
MP-V5	2.38E-10	5.09E-07	6.2

Supplementary Table 3. Summary of induced and uninduced mutagenesis levels for all designed MPs. All MPs were tested using the rifampin resistance assay to assess their relative mutagenic load under uninduced (glucose) and induced (arabinose) conditions. The viability of the MP-carrying strains under the induced conditions (as a percentage of the viability of the strain without an MP) is also shown. Ideal MPs show low background and high induced mutagenesis, with only moderate reductions in viability.

Source organism	Gene(s)	Fraction Rif^R		μ_{bp} (bp^{-1} generation^{-1})			vs MP6	Dynamic	Reference
		- MP	+ MP	- MP	+ MP	Fold	(%)	range*	
Ec	dam	9.00E-09	3.00E-07	6.16E-11	2.05E-09	0	0	-	22
Ec	dnaE173	5.00E-09	7.20E-06	3.42E-11	4.93E-08	1440	1	24	4
Ec	dnaQ926	3.00E-08	3.47E-04	2.05E-10	2.38E-06	11567	38	-	12
Ec	mutD5	3.00E-08	5.70E-05	2.05E-10	3.90E-07	1900	6	-	12
Sc	mag1	5.13E-08	3.61E-06	3.51E-10	2.47E-08	70	0	-	23
Sc	mag1	1.00E-08	2.00E-06	6.85E-11	1.37E-08	200	0	200	28
Ec	dinB	4.00E-08	4.55E-06	2.74E-10	3.11E-08	114	0	114	29
Ec	dnaE (K655Y)	1.00E-08	8.45E-06	6.85E-11	5.78E-08	845	1	-	16
Hs	AID	1.30E-08	1.03E-07	8.90E-11	7.05E-10	8	0	8	30
Rn	APOBEC1	2.53E-08	1.23E-05	1.73E-10	8.42E-08	486	1	14	31
Rn	APOBEC2	2.53E-08	2.50E-08	1.73E-10	1.71E-10	1	0	-	31
Hs	AID	2.53E-08	1.66E-07	1.73E-10	1.14E-09	7	0	-	31
Hs	APOBEC3C	2.53E-08	2.93E-07	1.73E-10	2.01E-09	12	0	-	31
Hs	APOBEC3G	2.53E-08	2.70E-07	1.73E-10	1.85E-09	11	0	-	31
Ec	mutH (E56A)	4.40E-08	8.74E-06	3.01E-10	5.98E-08	199	1	-	3
Ec	mutH (K116E)	4.40E-08	8.40E-06	3.01E-10	5.75E-08	191	1	-	3
Ec	mutH (K79E)	4.40E-08	7.00E-06	3.01E-10	4.79E-08	159	1	-	3
Ec	mutH (E77A)	4.40E-08	6.94E-06	3.01E-10	4.75E-08	158	1	-	3
Ec	mutH (D70A)	4.40E-08	6.88E-06	3.01E-10	4.71E-08	156	1	-	3
Ec	mutH CΔ5	4.40E-08	9.10E-07	3.01E-10	6.23E-09	21	0	-	3
Ec	mutL (R95F/N302A)	4.40E-08	1.17E-05	3.01E-10	8.03E-08	267	1	-	3
Ec	mutL (R261H)	4.40E-08	1.04E-05	3.01E-10	7.13E-08	237	1	-	3
Ec	mutL (E29A)	4.40E-08	8.32E-06	3.01E-10	5.70E-08	189	1	-	3
Ec	mutL (K307A)	4.40E-08	6.75E-06	3.01E-10	4.62E-08	153	1	-	3
Ec	mutL (N302A)	4.40E-08	3.50E-06	3.01E-10	2.40E-08	80	0	-	3
Ec	mutL (R95F)	4.40E-08	1.27E-07	3.01E-10	8.69E-10	3	0	-	3
Ec	mutL (K159E)	4.40E-08	7.26E-06	3.01E-10	4.97E-08	165	1	-	3
Ec	mutL (R266E)	4.40E-08	6.05E-06	3.01E-10	4.14E-08	138	1	-	3
Ec	mutL (R177E)	4.40E-08	1.77E-06	3.01E-10	1.21E-08	40	0	-	3
Ec	mutL (I90R)	4.40E-08	1.56E-07	3.01E-10	1.07E-09	4	0	-	3
Ec	mutL (R237E)	4.40E-08	1.40E-07	3.01E-10	9.58E-10	3	0	-	3
Ec	mutL (G238A)	4.40E-08	5.40E-08	3.01E-10	3.70E-10	1	0	-	3
Ec	mutL (G238D)	4.40E-08	1.03E-05	3.01E-10	7.05E-08	234	1	-	3
Ec	mutL (I90E)	4.40E-08	1.70E-08	3.01E-10	1.16E-10	0	0	-	3
Ec	mutS (S668A/T669V)	4.40E-08	1.33E-05	3.01E-10	9.12E-08	303	1	-	3
Ec	mutS (K620M)	4.40E-08	1.21E-05	3.01E-10	8.31E-08	276	1	-	3
Ec	mutS (D693A)	4.40E-08	1.15E-05	3.01E-10	7.90E-08	262	1	-	3
Ec	mutS (E694Q)	4.40E-08	1.13E-05	3.01E-10	7.75E-08	257	1	-	3
Ec	mutS (E694A)	4.40E-08	8.84E-06	3.01E-10	6.05E-08	201	1	-	3
Ec	mutS (E694A/S668A/T669V)	4.40E-08	8.02E-06	3.01E-10	5.49E-08	182	1	-	3
Ec	mutS (D693N)	4.40E-08	5.58E-06	3.01E-10	3.82E-08	127	1	-	3
Ec	mutS (H760A)	4.40E-08	8.01E-07	3.01E-10	5.48E-09	18	0	-	3
Ec	mutS (H728A)	4.40E-08	4.48E-07	3.01E-10	3.07E-09	10	0	-	3
Ec	mutS (F596A)	4.40E-08	3.14E-07	3.01E-10	2.15E-09	7	0	-	3
Ec	mutS (S612A)	4.40E-08	3.70E-08	3.01E-10	2.53E-10	1	0	-	3
Ec	mutS (F36A)	4.40E-08	1.06E-05	3.01E-10	7.26E-08	241	1	-	3
Ec	mutS (E38Q)	4.40E-08	4.19E-06	3.01E-10	2.87E-08	95	0	-	3
Ec	mutS (D162R/E164R)	4.40E-08	4.30E-06	3.01E-10	2.94E-08	98	0	-	3
Ec	mutS (R163E)	4.40E-08	3.90E-08	3.01E-10	2.67E-10	1	0	-	3
Ec	mutS (R197E/R198E)	4.40E-08	1.11E-05	3.01E-10	7.61E-08	253	1	-	3
Ec	mutS (R197E/R198E/R199E)	4.40E-08	6.15E-06	3.01E-10	4.21E-08	140	1	-	3
Ec	mutS (E177A)	4.40E-08	1.10E-05	3.01E-10	7.50E-08	249	1	-	3
Ec	mutS (T115A)	4.40E-08	7.99E-06	3.01E-10	5.47E-08	182	1	-	3
Ec	dam	3.00E-08	2.00E-06	2.05E-10	1.37E-08	67	0	-	5
Ec	emrR	3.00E-08	5.00E-06	2.05E-10	3.42E-08	167	1	-	5
Ec	mutS*	3.00E-08	3.00E-07	2.05E-10	2.05E-09	10	0	-	5
Ec	seqA	3.00E-08	8.00E-07	2.05E-10	5.48E-09	27	0	-	5
Ec	dinB	3.00E-08	3.00E-07	2.05E-10	2.05E-09	10	0	-	5
Pa	nfxB	8.00E-08	3.50E-06	5.48E-10	2.40E-08	44	0	-	32
Ll	dnaN	8.00E-08	9.80E-08	5.48E-10	6.71E-10	1	0	-	32
Ll	dnaA	8.00E-08	1.90E-07	5.48E-10	1.30E-09	2	0	-	32
Ll	uvrA, ysjE	8.00E-08	3.90E-08	5.48E-10	2.67E-10	0	0	-	32
Ll	uvrA	8.00E-08	1.10E-07	5.48E-10	7.53E-10	1	0	-	32
Ll	rnhA, sipL, purR	8.00E-08	4.80E-08	5.48E-10	3.29E-10	1	0	-	32
Ll	rnhA, sipL	8.00E-08	6.70E-08	5.48E-10	4.59E-10	1	0	-	32
Ll	rnhA	8.00E-08	1.40E-08	5.48E-10	9.58E-11	0	0	-	32
Ec	polB	2.20E-08	2.32E-06	1.51E-10	1.59E-08	105	0	116	26
Ec	polB Q779V	2.20E-08	6.14E-08	1.51E-10	4.20E-10	3	0	-	26
Ec	polB Δ780-783	2.20E-08	5.93E-08	1.51E-10	4.06E-10	3	0	-	26
Ec	polB (D156A)	2.20E-08	4.73E-04	1.51E-10	3.23E-06	21478	52	-	26
Ec	uvrAB	7.90E-09	5.40E-08	5.41E-11	3.70E-10	7	0	-	33
Ec	uvrABC	7.90E-09	1.90E-07	5.41E-11	1.30E-09	24	0	-	33

Hs	AID	1.00E-08	1.40E-07	6.85E-11	9.58E-10	14	0	-	25
Hs	AID (K10E/E156G)	1.00E-08	6.30E-07	6.85E-11	4.31E-09	63	0	-	25
Hs	AID (K34E/K160E)	1.00E-08	4.20E-07	6.85E-11	2.88E-09	42	0	-	25
Hs	AID	2.40E-08	4.90E-07	1.64E-10	3.35E-09	20	0	-	34
Hs	AID-3FL	2.40E-08	3.70E-07	1.64E-10	2.53E-09	15	0	-	34
Hs	AID-3GL	2.40E-08	1.60E-07	1.64E-10	1.10E-09	7	0	-	34
Hs	AAG	1.00E-09	2.00E-07	6.85E-12	1.37E-09	200	0	1	24
Hs	AAG(Y127I/H136L)	1.00E-09	1.15E-05	6.85E-12	7.87E-08	11500	1	4	24
Ec	nrdAB	1.00E-08	8.00E-08	6.85E-11	5.48E-10	8	0	-	7
Ec	nrdEF	1.00E-08	3.50E-07	6.85E-11	2.40E-09	35	0	-	7
Ec	nrdA(H59A)B	1.00E-08	3.80E-07	6.85E-11	2.60E-09	38	0	-	7
Hs	APOBEC3G	2.60E-07	2.40E-06	1.78E-09	1.64E-08	9	0	-	35
Hs	APOBEC3G (E259Q)	2.60E-07	3.60E-07	1.78E-09	2.46E-09	1	0	-	35
Hs	APOBEC3G (E254R)	2.60E-07	3.50E-06	1.78E-09	2.40E-08	13	0	-	35
Hs	APOBEC3G (R313E)	2.60E-07	2.60E-07	1.78E-09	1.78E-09	1	0	-	35
Hs	APOBEC3G (R320E)	2.60E-07	5.80E-07	1.78E-09	3.97E-09	2	0	-	35
Hs	APOBEC3G (R313E/R320E)	2.60E-07	3.10E-07	1.78E-09	2.12E-09	1	0	-	35
Hs	APOBEC3G (R374E)	2.60E-07	5.50E-07	1.78E-09	3.77E-09	2	0	-	35
Hs	APOBEC3G (R376E)	2.60E-07	1.90E-06	1.78E-09	1.30E-08	7	0	-	35
Hs	APOBEC3G (R374E/R376E)	2.60E-07	6.90E-07	1.78E-09	4.72E-09	3	0	-	35
Hs	APOBEC3G (R213E)	2.60E-07	4.40E-07	1.78E-09	3.01E-09	2	0	-	35
Hs	APOBEC3G (R215E)	2.60E-07	3.20E-07	1.78E-09	2.19E-09	1	0	-	35
Hs	APOBEC3G (R213E/215E)	2.60E-07	2.90E-07	1.78E-09	1.99E-09	1	0	-	35
Ec	dnaQ926, umuD', umuC, recA730	2.83E-09	9.38E-06	1.94E-11	6.42E-08	3320	1	78	36
Ec	dnaQ926	2.83E-09	1.44E-05	1.94E-11	9.85E-08	5087	2	355	This work
Ec	dnaQ926, dam	2.83E-09	3.88E-05	1.94E-11	2.66E-07	13723	4	15	This work
Ec	dnaQ926, dam, seqA	2.83E-09	6.39E-05	1.94E-11	4.38E-07	22610	7	1604	This work
Ec, PBS2, Pm	dnaQ926, dam, seqA, ugi, cda1	2.83E-09	2.93E-04	1.94E-11	2.01E-06	103749	32	419	This work
Ec, PBS2, Pm	dnaQ926, dam, seqA, emrR, ugi, cda1	2.83E-09	9.11E-04	1.94E-11	6.24E-06	322414	100	34941	This work

Supplementary Table 4. Comparison of MP1-MP6 with previously described mutator plasmids. In each case, the mutator genes are listed with the source organism(s): *Ec = Escherichia coli; Sc = Saccharomyces cerevisiae; Hs = Homo sapiens; Rn = Rattus norvegicus; Pa = Pseudomonas aeruginosa; Ll = Lactococcus lactis; PBS2 = Bacillus subtilis phage PBS2; Pm = Petromyzon marinus*. In all cases, the fraction of cells showing rifampin resistance (Rif^R) was used to calculate μ_{bp} as described in the methods section, using R=77 sites, $N=10^8$, and $N_0 = 1.5\text{x}10^7$ to approximate the levels as compared to the MP1-6 series. The fold increase in mutagenesis is shown for each MP (defined as the ratio of the mutagenesis in the strain without the MP vs. in the strain with the MP). All MPs are compared to MP6 in total mutagenesis efficiency. *In cases where the MP was inducible, the dynamic range represents the fold increase between the uninduced and induced states for strains carrying the MP.

Antibiotic	MIC (ug/mL)
carbenicillin	2 - 25
cefotaxime	0.016 - 0.25
fosfomycin	0.125 - 8
kanamycin	0.25 - 8
metronidazole	8 - 32
norfloxacin	0.016 - 0.125
rifampin	0.5 - 16
spectinomycin	8 - 64
streptomycin	1 - 16
tetracycline	0.5 - 8

Supplementary Table 5. Minimum inhibitory concentrations (MICs) for selected antibiotics. All data regarding *E. coli* MICs was tabulated from the Antimicrobial Index Knowledgebase (http://antibiotics.toku-e.com).

	CRB	CTX	FOS	KAN	MTX	RIF	SPC	STR	TET
no MP	0.00E+00	0.00E+00	1.10E-05	5.78E-06	2.22E-07	5.56E-08	0.00E+00	6.67E-07	0.00E+00
MP1	9.57E-06	0.00E+00	9.17E-04	1.84E-04	6.47E-01	1.26E-05	1.91E-07	5.68E-06	0.00E+00
MP4	1.37E-05	0.00E+00	6.97E-03	6.56E-03	1.52E-06	6.17E-05	1.14E-05	1.56E-05	5.97E-06
MP6	7.39E-04	0.00E+00	2.78E-02	1.11E-01	1.50E-04	7.17E-04	2.55E-04	6.22E-04	1.15E-04
2AP	1.17E-07	2.80E-06	4.83E-04	1.53E-03	2.03E-05	1.03E-05	2.50E-07	3.17E-07	0.00E+00
EMS	0.00E+00	1.67E-07	3.75E-04	8.17E-04	1.10E-04	6.68E-05	0.00E+00	1.33E-06	0.00E+00
MNNG	0.00E+00	3.67E-07	1.22E-04	5.33E-04	3.22E-05	5.56E-06	3.67E-05	2.44E-07	0.00E+00
UV	0.00E+00	0.00E+00	5.00E-07	7.49E-06	0.00E+00	0.00E+00	0.00E+00	2.89E-08	0.00E+00
XL1-Blue	0.00E+00	5.40E-06	0.00E+00	8.66E-07	4.82E-01	0.00E+00	0.00E+00	0.00E+00	9.44E-01
XL1-Red	0.00E+00	0.00E+00	1.42E-03	2.62E-04	9.84E-01	3.62E-05	3.81E-06	3.60E-05	1.10E-04

Supplementary Table 6. Comparison of developed MPs to chemical mutagens, UV light and XL1-Red. The fraction of cells resistant to each antibiotic upon mutagenic treatment is shown for all tested antibiotics. No resistance was observed for norfloxacin using any MP, chemical mutagen, or strain. All MPs, chemical mutagens, and UV light treatments used *E. coli* MG1655 *ΔrecA::apra*. CRB, 50 μg/mL carbenicillin; CTX, 5 μg/mL cefotaxime; FOS, 100 μg/mL fosfomycin; KAN, 30 μg/mL kanamycin, MTX, 100 μg/mL metronidazole; RIF, 100 μg/mL rifampin; SPC, 100 μg/mL spectinomycin; STR, 50 μg/mL streptomycin; TET, 10 μg/mL tetracycline. We note that MP1 (*recA730*), XL1-Blue (*recA1*), and XL1-Red (wt *recA*) are all proficient at recombination, a known requirement for high-level resistance to metronidazole[37]. Additionally, XL1-Blue and XL1-Red are both inherently resistant to tetracycline, explaining the observed high incidence of resistance.

Supplementary Notes

Supplementary Note 1. MP6 optimization

We retained native bacterial ribosome-binding sites (RBSs) upstream of the ORFs for four of the six MP6 genes. The exceptions are *dam*, which natively lacks a canonical RBS in the *E. coli* genome, and *cda1*, which derives from the eukaryote *P. marinus* and thus does not use a bacterial Shine-Dalgarno sequence. In an attempt to further enhance mutational potency by modulating the expression of *dnaQ926*, *dam*, *seqA*, *emrR*, *ugi*, and *cda1*, we varied RBS upstream of each of these six genes by individually mutating them to fully complement the 16S rRNA, and resulting in optimal transcript translation. Interestingly, strengthening the RBSs upstream of each of the six genes generally reduced the potency of the MP, with the exception of the *seqA* and *dnaQ926* RBSs (Supplementary Tables 2 and 3). Strengthening the *seqA* RBS proved highly lethal under induced conditions, likely as a consequence of impeded genomic replication (Supplementary Tables 2 and 3). Increasing the strength of the *dnaQ926* RBS enhanced the mutagenic potency of the MP by 4-fold under induced conditions, concomitant with a minor increase in background mutagenesis, but was more toxic to bacteria as evidenced by a greater loss of viability under induced conditions (Supplementary Tables 2 and 3). Additionally, measurement of the mutation rate of the resulting MP became irreproducible, consistent with MP instability under these conditions. These findings together suggest that additional mutagenic potency gains beyond that of MP6 may result in error catastrophe and reduced MP stability.

Supplementary Note 2. ***LacZ* reversion analysis**

Strains CSH101 to CSH106 each carry different nonfunctional missense mutants of *lacZ* at codon 461 (natively encoding glutamic acid) on the *F'* episome. If a mutation reverts the nonfunctional codon to a glutamic acid, the strain can synthesize functional LacZ and survives using lactose as the only carbon source. These six strains are designed to report on all 12 possible mutations using this codon reversion. Using these strains, we observed that MP1 had the most narrow episomal mutational spectrum, with a moderate bias towards G:C → A:T (CSH102) and A:T → G:C (CSH106) substitutions (Supplementary Figure 11). MP4 showed an improved distribution of mutations, with a still moderate preference for A:T → G:C (CSH106) substitutions (Supplementary Figure 10). MP6 showed a near equal distribution of mutations in all six strains, with the exception of A:T → C:G (CSH101) substitutions which were detected at 10- to 100-fold lower levels than the other substitutions (Supplementary Figure 10). Taken together, these results suggest that MP6 generally outperforms the other MPs and XL1-Red based on both the frequency of *lacZ* reversion as well as the breadth of mutation types detected by these strains.

Importantly, the mutational potency and spectra using the episomal *lacZ* reversion assays were in agreement with those from the rifampin resistance assays (Fig. 4A-E, Supplementary Figure 11). These results establish the ability of the MPs to affect a wide variety of mutations in both genomic and episomal DNA.

Supplementary References

1. Horst, J.P., Wu, T.H. & Marinus, M.G. Escherichia coli mutator genes. *Trends in microbiology* **7**, 29-36 (1999).
2. Aronshtam, A. & Marinus, M.G. Dominant negative mutator mutations in the mutL gene of Escherichia coli. *Nucleic acids research* **24**, 2498-2504 (1996).
3. Junop, M.S., Yang, W., Funchain, P., Clendenin, W. & Miller, J.H. In vitro and in vivo studies of MutS, MutL and MutH mutants: correlation of mismatch repair and DNA recombination. *DNA repair* **2**, 387-405 (2003).
4. Maki, H., Mo, J.Y. & Sekiguchi, M. A strong mutator effect caused by an amino acid change in the alpha subunit of DNA polymerase III of Escherichia coli. *The Journal of biological chemistry* **266**, 5055-5061 (1991).
5. Yang, H., Wolff, E., Kim, M., Diep, A. & Miller, J.H. Identification of mutator genes and mutational pathways in Escherichia coli using a multicopy cloning approach. *Molecular microbiology* **53**, 283-295 (2004).
6. Wu, T.H. & Marinus, M.G. Dominant negative mutator mutations in the mutS gene of Escherichia coli. *Journal of bacteriology* **176**, 5393-5400 (1994).
7. Gon, S., Napolitano, R., Rocha, W., Coulon, S. & Fuchs, R.P. Increase in dNTP pool size during the DNA damage response plays a key role in spontaneous and induced-mutagenesis in Escherichia coli. *Proceedings of the National Academy of Sciences of the United States of America* **108**, 19311-19316 (2011).
8. Troll, C. et al. The mutagenic footprint of low-fidelity Pol I ColE1 plasmid replication in E. coli reveals an extensive interplay between Pol I and Pol III. *Current genetics* **60**, 123-134 (2014).
9. Balashov, S. & Humayun, M.Z. Specificity of spontaneous mutations induced in mutA mutator cells. *Mutation research* **548**, 9-18 (2004).
10. Stratagene Overcome mutational bias. *Strategies* **17**, 20–21 (2004).
11. Lai, Y.P., Huang, J., Wang, L.F., Li, J. & Wu, Z.R. A new approach to random mutagenesis in vitro. *Biotechnology and bioengineering* **86**, 622-627 (2004).
12. Fijalkowska, I.J. & Schaaper, R.M. Mutants in the Exo I motif of Escherichia coli dnaQ: defective proofreading and inviability due to error catastrophe. *Proceedings of the National Academy of Sciences of the United States of America* **93**, 2856-2861 (1996).
13. Fijalkowska, I.J., Dunn, R.L. & Schaaper, R.M. Genetic requirements and mutational specificity of the Escherichia coli SOS mutator activity. *Journal of bacteriology* **179**, 7435-7445 (1997).
14. Wijesinghe, P. & Bhagwat, A.S. Efficient deamination of 5-methylcytosines in DNA by human APOBEC3A, but not by AID or APOBEC3G. *Nucleic acids research* **40**, 9206-9217 (2012).
15. Lada, A.G. et al. Mutator effects and mutation signatures of editing deaminases produced in bacteria and yeast. *Biochemistry. Biokhimiia* **76**, 131-146 (2011).
16. Strauss, B.S., Roberts, R., Francis, L. & Pouryazdanparast, P. Role of the dinB gene product in spontaneous mutation in Escherichia coli with an impaired replicative polymerase. *Journal of bacteriology* **182**, 6742-6750 (2000).
17. Wechsler, J.A. et al. Isolation and characterization of thermosensitive Escherichia coli mutants defective in deoxyribonucleic acid replication. *Journal of bacteriology* **113**, 1381-1388 (1973).
18. Yang, H., To, K.H., Aguila, S.J. & Miller, J.H. Metagenomic DNA fragments that affect Escherichia coli mutational pathways. *Molecular microbiology* **61**, 960-977 (2006).
19. Pham, P.T., Zhao, W. & Schaaper, R.M. Mutator mutants of Escherichia coli carrying a defect in the DNA polymerase III tau subunit. *Molecular microbiology* **59**, 1149-1161 (2006).
20. Ahluwalia, D., Bienstock, R.J. & Schaaper, R.M. Novel mutator mutants of E. coli nrdAB ribonucleotide reductase: insight into allosteric regulation and control of mutation rates. *DNA repair* **11**, 480-487 (2012).

21. Dahlgren, A. & Ryden-Aulin, M. A novel mutation in ribosomal protein S4 that affects the function of a mutated RF1. *Biochimie* **82**, 683-691 (2000).
22. Herman, G.E. & Modrich, P. Escherichia coli K-12 clones that overproduce dam methylase are hypermutable. *Journal of bacteriology* **145**, 644-646 (1981).
23. Glassner, B.J., Rasmussen, L.J., Najarian, M.T., Posnick, L.M. & Samson, L.D. Generation of a strong mutator phenotype in yeast by imbalanced base excision repair. *Proceedings of the National Academy of Sciences of the United States of America* **95**, 9997-10002 (1998).
24. Klapacz, J. et al. Frameshift mutagenesis and microsatellite instability induced by human alkyladenine DNA glycosylase. *Molecular cell* **37**, 843-853 (2010).
25. Wang, M., Yang, Z., Rada, C. & Neuberger, M.S. AID upmutants isolated using a high-throughput screen highlight the immunity/cancer balance limiting DNA deaminase activity. *Nature structural & molecular biology* **16**, 769-776 (2009).
26. Al Mamun, A.A. Elevated expression of DNA polymerase II increases spontaneous mutagenesis in Escherichia coli. *Mutation research* **625**, 29-39 (2007).
27. Luan, G., Cai, Z., Li, Y. & Ma, Y. Genome replication engineering assisted continuous evolution (GREACE) to improve microbial tolerance for biofuels production. *Biotechnology for biofuels* **6**, 137 (2013).
28. Posnick, L.M. & Samson, L.D. Imbalanced base excision repair increases spontaneous mutation and alkylation sensitivity in Escherichia coli. *Journal of bacteriology* **181**, 6763-6771 (1999).
29. Ren, L., Al Mamun, A.A. & Humayun, M.Z. The mutA mistranslator tRNA-induced mutator phenotype requires recA and recB genes, but not the derepression of lexA-regulated functions. *Molecular microbiology* **32**, 607-615 (1999).
30. Petersen-Mahrt, S.K., Harris, R.S. & Neuberger, M.S. AID mutates E. coli suggesting a DNA deamination mechanism for antibody diversification. *Nature* **418**, 99-103 (2002).
31. Harris, R.S., Petersen-Mahrt, S.K. & Neuberger, M.S. RNA editing enzyme APOBEC1 and some of its homologs can act as DNA mutators. *Molecular cell* **10**, 1247-1253 (2002).
32. Gabrovsky, V., Yamamoto, M.L. & Miller, J.H. Mutator effects in Escherichia coli caused by the expression of specific foreign genes. *Journal of bacteriology* **187**, 5044-5048 (2005).
33. Hasegawa, K., Yoshiyama, K. & Maki, H. Spontaneous mutagenesis associated with nucleotide excision repair in Escherichia coli. *Genes to cells : devoted to molecular & cellular mechanisms* **13**, 459-469 (2008).
34. Kohli, R.M. et al. A portable hot spot recognition loop transfers sequence preferences from APOBEC family members to activation-induced cytidine deaminase. *The Journal of biological chemistry* **284**, 22898-22904 (2009).
35. Shindo, K. et al. A Comparison of Two Single-Stranded DNA Binding Models by Mutational Analysis of APOBEC3G. *Biology* **1**, 260-276 (2012).
36. Carlson, J.C., Badran, A.H., Guggiana-Nilo, D.A. & Liu, D.R. Negative selection and stringency modulation in phage-assisted continuous evolution. *Nature chemical biology* **10**, 216-222 (2014).
37. Yeung, T.C., Beaulieu, B.B., Jr., McLafferty, M.A. & Goldman, P. Interaction of metronidazole with DNA repair mutants of Escherichia coli. *Antimicrobial agents and chemotherapy* **25**, 65-70 (1984).
